# *Grpr* expression defines a population of superficial dorsal horn vertical cells that have a role in both itch and pain

**DOI:** 10.1097/j.pain.0000000000002677

**Published:** 2022-05-11

**Authors:** Erika Polgár, Allen C. Dickie, Maria Gutierrez-Mecinas, Andrew M. Bell, Kieran A. Boyle, Raphaëlle Quillet, Elisha Ab Rashid, Ross A. Clark, Morgan T. German, Masahiko Watanabe, John S. Riddell, Andrew J. Todd

**Affiliations:** a Spinal Cord Group, School of Psychology and Neuroscience, University of Glasgow, Glasgow, United Kingdom; b Department of Anatomy, Hokkaido University School of Medicine, Sapporo, Japan

**Keywords:** Gastrin-releasing peptide receptor, Vertical cell, Pain, Itch, Spinal cord

## Abstract

Neurons in the superficial dorsal horn that express the gastrin-releasing peptide receptor (GRPR) are strongly implicated in spinal itch pathways. However, a recent study reported that many of these correspond to vertical cells, a population of interneurons that are believed to transmit nociceptive information. In this study, we have used a GRPR^CreERT2^ mouse line to identify and target cells that possess *Grpr* mRNA. We find that the GRPR cells are highly concentrated in lamina I and the outer part of lamina II, that they are all glutamatergic, and that they account for ∼15% of the excitatory neurons in the superficial dorsal horn. We had previously identified 6 neurochemically distinct excitatory interneuron populations in this region based on neuropeptide expression and the GRPR cells are largely separate from these, although they show some overlap with cells that express substance P. Anatomical analysis revealed that the GRPR neurons are indeed vertical cells, and that their axons target each other, as well as arborising in regions that contain projection neurons: lamina I, the lateral spinal nucleus, and the lateral part of lamina V. Surprisingly, given the proposed role of GRPR cells in itch, we found that most of the cells received monosynaptic input from Trpv1-expressing (nociceptive) afferents, that the majority responded to noxious and pruritic stimuli, and that chemogenetically activating them resulted in pain-related and itch-related behaviours. Together, these findings suggest that the GRPR cells are involved in spinal cord circuits that underlie both pain and itch.

## Abstract 2

Supplemental Digital Content is Available in the Text.

Gastrin-releasing peptide receptor–expressing spinal interneurons have been implicated in itch. This study provides evidence that they also contribute to pain mechanisms.

## 1. Introduction

The superficial dorsal horn (SDH, laminae I-II) of the spinal cord is innervated by fine-diameter primary afferents that function as nociceptors, pruritoceptors, and thermoreceptors. Information conveyed by these afferents is transmitted through complex neuronal circuits, involving local neurons and descending axons, before being conveyed to the brain by projection neurons belonging to the anterolateral system (ALS).^10,60^

Although these projection neurons are concentrated in lamina I, most (∼99%) neurons in SDH are interneurons, and ∼75% of these are excitatory.^48^ A widely accepted classification scheme established by Grudt and Perl^22^ identified 3 distinctive classes among the excitatory interneurons in lamina II: vertical, radial, and transient central cells. Subsequent studies identified a pathway through which another set of excitatory interneurons (those expressing protein kinase Cγ, PKCγ) activate a circuit involving transient central cells, vertical cells, and lamina I projection neurons.^36,37^ It was proposed that vertical cells normally convey nociceptive input to lamina I projection neurons,^70^ and that in neuropathic states, loss of inhibitory input to the PKCγ cells allows low-threshold mechanoreceptive inputs to activate this circuit, thus conveying tactile information to a normally nociceptive pathway, and contributing to allodynia.^36^

Recent neurochemical studies have revealed several populations among the excitatory interneurons.^27,53,61^ We have identified 6 largely nonoverlapping classes of excitatory interneurons in the SDH. Five of these are defined by expression of cholecystokinin (CCK), neurotensin, neurokinin B (NKB), substance P (SP), and neuropeptide FF (NPFF), and the sixth by the presence of green fluorescent protein (GFP) in a GRP::GFP transgenic mouse line.^7,23,25,26^ These classes account for ∼75% of excitatory interneurons in laminae I-II and show good correspondence with transcriptomic populations.^27^ We reported that the SP and GRP–GFP populations include radial and transient central cells, respectively, whereas the CCK, neurotensin, and NKB populations overlap extensively with PKCγ cells in the inner part of lamina II and lamina III.^16,25^

Another population of excitatory interneurons that has attracted considerable attention consists of cells expressing the gastrin-releasing peptide receptor (GRPR). These are located in lamina I and outer lamina II, and there is strong evidence implicating them in itch. For example, mice lacking GRPR showed normal pain behaviours, but reduced pruritogen-evoked itch, while intrathecal administration of a gastrin-releasing peptide (GRP) agonist caused scratching that was blocked by administration of an antagonist.^57^ In addition, ablation of GRPR-expressing cells reduces itch, but not pain, behaviours.^58^ This has led to the suggestion that GRP released from other excitatory interneurons acts on the GRPR cells, which therefore represent "tertiary pruritoceptors” in the spinal itch circuit.^40,44^ However, a recent study^34^ reported that GRPR neurons, identified in a GRPR::GFP transgenic mouse line, resembled vertical cells, which (as noted above) are believed to be part of a spinal pain pathway.^36^ The aim of this study was to determine the relationship of GRPR neurons to other neurochemical populations that we have defined and to test the hypothesis that these cells transmit both noxious and pruritic information.

## 2. Methods

Experiments were approved by the Ethical Review Process Applications Panel of the University of Glasgow and were performed in accordance with the UK Animals (Scientific Procedures) Act 1986.

### 2.1. Animals and operative procedures

We used a mouse line (GRPR^CreERT2^) in which an insert coding for a fusion protein consisting of codon-improved Cre recombinase (iCre) and mutated estrogen receptor was located in the GRPR locus.^41^ Bicistronic expression ensured that both iCre and GRPR were expressed simultaneously. In some cases, the GRPR^CreERT2^ mice were crossed with the Ai9 reporter line (Jackson Laboratory; Stock number 007909) in which Cre-mediated excision of a STOP cassette results in expression of the red fluorescent protein tdTomato. We also crossed the GRPR^CreERT2^ line with Ai9 and GRP::GFP mice to generate a GRPR^CreERT2^;Ai9;GRP::GFP cross.

Mice of both sexes, weighing between 14 and 29 g, were used for the study. The animals were between 5 and 14-week-old at the time of tissue harvest (for anatomy), electrophysiological recording, or behavioural testing. All mice, apart from those used for in situ hybridisation, received intraperitoneal injections of tamoxifen. In preliminary experiments, we had tested the effect of different doses of tamoxifen (2, 4, 6, and 9 mg) on GRPR^CreERT2^;Ai9 mice and found that the numbers of tdTomato cells in laminae I-II increased with increasing dose up to 6 mg, but that there was no further increase at 9 mg. We therefore used a 6-mg dose (2 intraperitoneal injections of 3 mg given on consecutive days) in most experiments. For those mice that had received intraspinal injection of viral vectors, tamoxifen was given at least 2 days after the operation. In all cases, animals survived for at least 4 days (and in most cases at least 1 week) after tamoxifen administration to allow adequate recombination. Because the GRPR gene is located on the X chromosome, experiments involving GRPR^CreERT2^ mice that required maximal capture used either male animals that were hemizygous or females that were homozygous for the mutated allele. This was the case for counts to estimate the proportion of neurons that were GRPR+ and for behavioural experiments that involved chemogenetic activation.

Intraspinal injections of viral vectors coding for Cre-dependent constructs were performed as described previously.^16,29,46^ In brief, mice were anaesthetised with isoflurane (∼1.5%) and received injections into the lumbar spinal cord. These were targeted into the L3 and L5 segments on one side, the L3, L4, and L5 segments on one side, or the L3 segment on both sides. Injections into the L3 and L5 segments were made through the spaces between the T12-T13 and T13-L1 vertebrae, respectively. For the L4 segment, a small hole was drilled through the lamina of T13 to provide access. Injections were made 400 μm lateral to the midline at a depth of 300 μm below the cord surface, with a flow rate of 30 nL per minute. After each injection, the pipette was left in place for 5 minutes to minimise leakage back up the track. Details of viruses are provided in Table 1 and a description of individual operations appears below. All animals that underwent surgical procedures received perioperative analgesia (buprenorphine 0.05 mg/kg and carprofen 5 mg/kg s.c.).

**Table 1:** Adeno-associated virus vectors.

	Serotype	Promoter	Construct(s)	Source	Catalogue number	Details of injection	
						Number of GCs	Volume
AAV.flex.eGFP	AAV2	hSyn1	eGFP	VVF Zurich	v115-2	2.0 × 10^8^	300 nL
AAV.flex.eGFP	AAV8	CAG	eGFP	VVF Zurich	v158-8	2.58 × 10^8^	300 nL
AAV.flex.tdTomato	AAV1	CAG	tdTomato	PennCore	cs1038	9.21 × 10^7^	300 nL
AAV.flex.tdTomato_Syp-eGFP	AAV1	hSyn1	tdTomato, Syp-eGFP	VVF Zurich	v155-1	5.4 × 10^8^	300 nL
AAV-EF1a-BbTagBY (AAV-BB1)	AAV9	hEF1a	TagBFP, YFP	Addgene	45185	7.55 × 10^7^	500 nL
AAV-EF1a-BbChT (AAV-BB2)	AAV9	hEF1a	mTFP, mCherry	Addgene	45186	7.44 × 10^7^	500 nL
AAV.DIO.dAPEX2	AAV1	hEF1a	Mutated soybean ascorbate peroxidase 1 dAPEX2	VVF Zurich	V568-1	2.07 × 10^9^	300 nL
AAV.flex.hM3D(Gq)	AAV2	hSyn1	hM3Dq-mCherry	VVF Zurich	v89-2	3.84 × 10^8^	300 nL

The values in columns 7 and 8 refer to individual injections; in most cases, mice received more than 1 injection into different segments and/or into different sides of the same segment. GCs, gene copies; eGFP, enhanced green fluorescent protein; mTFP, membrane-targeted teal fluorescent protein.

Tissue for fluorescence in situ hybridization experiments was obtained from mice that had been decapitated under deep isoflurane anaesthesia, and this was rapidly frozen on dry ice. For electrophysiological experiments, mice were anaesthetised with pentobarbitone (20 mg i.p.) and perfused through the vascular system with ice-cold dissection solution. They were then decapitated, and the spinal cord was rapidly removed. All other mice, including those used for behavioural experiments (see below), underwent perfusion fixation under deep terminal anaesthesia. They were anaesthetised with pentobarbitone as described above and perfused through the heart with fixative. Unless otherwise stated, this contained 4% freshly depolymerised formaldehyde in phosphate buffer, and spinal cord tissue was rapidly dissected out and postfixed at 4°C for 2 hours.

For convenience, we refer to cells that expressed Cre in the GRPR^CreERT2^ line (detected either with reporter crosses or by the viral strategy) as GRPR cells.

### 2.2. Fluorescence in situ hybridisation

Fluorescence in situ hybridisation was performed with RNAscope probes and RNAscope fluorescent multiplex reagent kit 320850 (ACD Bio-Techne; Newark, CA 94560). Fresh frozen lumbar spinal cords from 4 wild-type mice and 2 GRPR^CreERT2^;Ai9 mice (homozygous for GRPR^CreERT2^) were embedded in optimal cutting temperature (OCT) medium and cut into transverse 12-μm thick sections with a cryostat (Leica CM1950; Leica, Milton Keynes, United Kingdom). The sections were mounted onto Superfrost Plus slides (48311-703; VWR; Lutterworth, United Kingdom) such that there was a gap of at least 36 μm in the rostrocaudal axis between sections. Reactions were performed according to the manufacturer's recommended protocol. Details of the probes are listed in Table 2. Sections from the wild-type mice were reacted with probes for *Grpr* and *Slc17a6* (4 mice). Sections from the GRPR^CreERT2^;Ai9 mice were reacted with probes for *Grpr* and *iCre*. Sections from the wild-type mice were also reacted for *Grpr* and either *Tac1* or *Gad1* (3 mice per reaction). Positive and negative control probes were also tested on other sections, as described previously.^25^ Sections were mounted with ProLong Glass antifade medium with NucBlue (Hoechst 33342; Thermo Fisher Scientific, Paisley, United Kingdom). The full thickness of the section was scanned with a Zeiss LSM 710 confocal microscope equipped with Argon multiline, 405-nm diode, 561-nm solid state, and 633-nm HeNe lasers, through a 40× oil immersion lens (numerical aperture, NA, 1.3), and tile scanning was used to cover the whole of laminae I-II.

**Table 2:** RNAscope probes used in this study.

Probe	Protein/peptide	Channel numbers	Catalogue numbers	Z-pair number	Target region
Grpr	GRPR	3	317871	20	463-1596
Slc17a6	VGLUT2	2	319171	20	1986-2998
Gad1	Glutamate decarboxylase (GAD) 67	1	400951	15	62-3113
iCre	Codon-improved Cre recombinase	2	423321	16	2-1028
Tac1	Substance P	1	410351	15	20-1034
RNAscope multiplex positive control (Polr2a, Ppib, and Ubc)	Polr2a: DNA-directed RNA polymerase II subunit RPB1; Ppib: Peptidyl-prolyl cis-trans isomerase B; and Ubc: Polyubiquitin-C	1,2,3	320881	20 15 n/a	2802-3678 98-856 34-860
RNAscope multiplex negative control (dapB)	dapB: 4-Hydroxy-tetrahydrodipicolinate reductase (derived from *B. subtilis*)	1,2,3	320871	10	414-862

Cells counts to determine the degree of colocalization between *Grpr* and *Gad1* or *Tac1* transcripts were performed manually using Neurolucida for Confocal software (MBF Bioscience, Williston, VT). The entire z-stack was examined, and transcripts belonging to a cell were judged as those distributed either within the nucleus or immediately adjacent to it. Cells were defined as positive for expression of a given gene if they contained 4 or more transcripts. For the analysis of the relationship between *Grpr* and *Slc17a6* or *iCre* transcripts, a quantitative analysis of transcript numbers was conducted using the cell detection and subcellular object features of QuPath software.^4^ Single representative optical sections from the centre of the image stacks were used. Recognition and segmentation of individual nuclei was performed based on NucBlue staining, and an additional 2 μm perimeter was added to each nucleus to allow detection of perinuclear transcripts. Any areas with poor nuclear segmentation were manually excluded from the analysis. Detection thresholds were adjusted manually until the markup accurately reflected the transcript distribution; then, a transcript count for each individual cell was obtained.

### 2.3. General features of immunohistochemistry

Multiple labelling fluorescence immunohistochemistry was performed as described previously.^7,25^ Transverse or sagittal sections 60 μm thick were cut from lumbar spinal cord segments with a vibrating blade microtome (Leica VT1000 or VT1200). Sections were incubated for 3 days in primary antibodies (listed in Table 3), which were diluted in phosphate-buffered saline (PBS) that contained 0.3% Triton X-100 and 5% normal donkey serum, and then overnight in appropriate species-specific secondary antibodies (Jackson ImmunoResearch, West Grove, PA), which were raised in donkey and conjugated to Alexa488, Alexa647, Rhodamine Red, Pacific Blue, or biotin. All secondary antibodies were diluted 1:500 (in the same diluent), apart from those conjugated to Rhodamine Red or Pacific Blue, which were diluted 1:100 and 1:200, respectively. Biotinylated secondary antibodies were revealed with Pacific Blue conjugated to avidin (1:1000; Life Technologies, Paisley, United Kingdom) or with a tyramide signal amplification method.^7^ After immunoreaction, sections were mounted in antifade medium and stored at −20°C. They were scanned with the Zeiss LSM 710 confocal microscope, through a 20× dry lens (NA 0.8) or 40× or 63× oil immersion lenses (NA 1.3 and 1.4, respectively) with the confocal aperture set to 1 Airy unit or less. Confocal scans were obtained with a 1 μm z-separation unless stated otherwise. Analyses were performed with Neurolucida for Confocal software.

**Table 3:** Antibodies used in this study.

Antigen	Species	Dilution	Source	Catalogue #	RRID
mCherry*	Rat	1:1000	Invitrogen	M11217	RRID:AB_2536611
mCherry*	Chicken	1:5000-1:10,000	Abcam	Ab205402	RRID:AB_2722769
mCherry*	Rabbit	1:1000	Abcam	Ab167453	RRID:AB_2571870
GFP	Chicken	1:1000	Abcam	Ab13970	RRID:AB_300798
mTFP	Rat	1:500	Kerafast	EMU103	
TagRFP†	Guinea pig	1:500	Kerafast	EMU107	
Pro-NPFF	Guinea pig	0.83 μg/mL	Gutierrez-Mecinas et al. (2019a)		RRID:AB_2783015
Pro-CCK	Rabbit	1:1000	Booker et al. (2017)		RRID:AB_2571674
Neurotensin	Rat	1:1000	Porteous et al. (2011)		RRID:AB_2314928
PPTB	Guinea pig	1:10,000	Kaneko et al. (1998)		RRID:AB_2783015
pERK	Rabbit	1:200	Cell Signaling Technology	9101	RRID:AB_331646
PKCγ	Guinea pig	1:1000	Yoshida et al. (2006)		RRID:AB_2571826
Pax2	Rabbit	1:1000	Life Technologies	716000	RRID:AB_2533990
NeuN	Guinea pig	1:1000	Synaptic Systems	266 004	RRID:AB_2619988

* mCherry antibodies recognise TdTomato.

† The tagRFP antibody recognises tagBFP.

### 2.4. Comparison of viral vs reporter strategies

To assess the efficiency of labelling in the GRPR^CreERT2^;Ai9 cross with that seen after spinal injection of adeno-associated virus (AAV) coding for Cre-dependent transgenes, we immunostained tissue from 3 GRPR^CreERT2^;Ai9 mice that had received intraspinal injections of AAV.flex.eGFP (either AAV2 or AAV8 serotypes). Transverse sections from segments containing the injection sites were reacted with antibodies against GFP, mCherry (rat antibody) and NeuN, and after incubation with secondary antibodies, the sections were counterstained with 4',6-diamidino-2-phenylindole (DAPI). A modification of the optical disector method^49^ was used to quantify neurons that expressed either tdTomato or GFP in the SDH. A 10 μm z-separation was set between reference and lookup sections, and all intervening sections were examined to reveal any neurons that might have been located between these 2 levels. In this way, we identified all neurons for which the bottom surface of the nucleus was located between the reference and lookup sections. Because this analysis revealed that the viral strategy labelled more cells (see below), we used this approach to determine the proportion of SDH neurons that were GRPR+. To do this, we used the disector technique to analyse tissue from 3 homozygous GRPR^CreERT2^ mice that had received intraspinal injections of AAV.flex.eGFP.

### 2.5. Relation of gastrin-releasing peptide receptor cells to other interneuron populations

We initially tested whether any of the GRPR cells were inhibitory by immunostaining sections from 2 GRPR^CreERT2^ mice that had received intraspinal injections of AAV.flex.eGFP with antibodies against Pax2 and NeuN. Sections were counterstained with DAPI, and the disector method was used to sample GFP-positive neurons. We then viewed the channel corresponding to Pax2 and noted its presence or absence in the selected GFP cells.

We also looked for overlap between the GRPR cells and other excitatory interneuron populations, defined by expression of NPFF, CCK, neurotensin, and NKB. These were revealed with antibodies against pro-NPFF, pro-CCK, neurotensin, and preprotachykinin B (PPTB), respectively. For each of these populations, we examined sections from 3 or 4 GRPR^CreERT2^ mice that had received intraspinal injection of AAV.flex.eGFP. Transverse sections were reacted with the following antibodies: (1) pro-NPFF and pro-CCK, (2) neurotensin and PKCγ, and (3) PPTB. The PKCγ antibody was used in the second series because it facilitates the identification of neurotensin-immunoreactive cells.^26^ We identified GFP+ neurons in confocal scans and determined whether they were immunoreactive for any of the peptides or peptide precursors.

In addition, we tested for overlap of the GRPR and GRP–GFP populations in sections from 3 GRPR^CreERT2^;Ai9;GRP::GFP mice. Sections were immunostained for GFP and mCherry (rat antibody). We identified tdTomato-positive cells and looked for the presence of GFP.

### 2.6. Morphology

We used the viral Brainbow method^11^ to reveal the somatodendritic morphology of GRPR cells. Three GRPR^CreERT2^ mice received 2 injections containing a mixture of AAV.BB1 and AAV.BB2 (Table 1), as described previously.^16^ After perfusion fixation, sagittal sections through the injected segments were reacted with antibodies against mCherry, mTFP, and tagRFP, which were revealed with Rhodamine Red, Alexa488, and Pacific Blue, respectively. The tagRFP antibody recognises tagBFP, which is expressed from AAV.BB2. Sections were scanned through the 63× lens with a z-spacing of 0.5 μm to generate z-series through the full thickness of the sections. Ten cells in lamina I or II were selected from scans obtained from each mouse. The selection was based on the following criteria: (1) relatively strong staining for at least one of the 3 fluorescent proteins, (2) location of the soma near the middle region of the z-axis so that most or all of the dendritic tree was likely to be contained within the section, and (3) the presence of a distinctive colour hue that was different from that of nearby cells, allowing unequivocal identification of the dendrites belonging to the selected cell.^16^ We also restricted the analysis to cells with a soma that was less than 40 μm below the dorsal border of the gray matter to exclude the relatively rare GRPR cells that are found in the deeper parts of the dorsal horn. Cell bodies and dendritic trees of the selected neurons were drawn with Neurolucida by following colour-coded processes originating from the soma. It was not possible to follow axons beyond their initial segments due to their very small diameter.

Because axons could not be followed in the Brainbow material, we reconstructed these from GRPR cells that had undergone patch-clamp recording and were filled with Neurobiotin (see below). The slices were fixed overnight in 4% formaldehyde, incubated in avidin conjugated to Alexa488, and immunostained with anti-mCherry (to reveal tdTomato). Slices were scanned with the confocal microscope through the 63× lens with 0.5 μm z-spacing, and axons were drawn from the resulting image stacks with Neurolucida. Finally, slices were resectioned at 60 μm thickness and the sections were immunostained to reveal PKCγ. They were rescanned with the confocal microscope, and the location of the PKCγ plexus was used to locate laminar boundaries.^31^

### 2.7. Synaptic connections between gastrin-releasing peptide receptor cells

During the course of this study, we noticed frequent contacts between axons and dendrites of GRPR cells. To investigate this, we examined sections from 3 GRPR^CreERT2^ mice that had received intraspinal injections consisting of a mixture of AAV.flex.tdTomato and AAV.flex.tdTomato_syp-eGFP. The sections were reacted with antibodies against GFP and mCherry (rabbit antibody). We also looked for contacts between axons belonging to GRPR cells that had undergone patch-clamp recording and nearby tdTomato-positive cells that had not been recorded from.

To confirm the presence of synapses between GRPR neurons, we used a viral vector to deliver peroxidase to these cells and then performed histochemistry and electron microscopy. Two GRPR^CreERT2^ mice received intraspinal injections of AAV.DIO.dAPEX2,^69^ which contains a Cre-dependent construct coding for a mutated soybean ascorbate peroxidase. After a 28-day survival period, the mice were reanaesthetised and perfused with a fixative containing 2.5% glutaraldehyde and 2% formaldehyde. The injected segments were postfixed for 24 hours and cut into transverse sections 60 μm thick with a Leica VT1200 microtome. These were rinsed in phosphate buffer (PB) containing 50 mM glycine and then placed in diaminobenzidine (DAB, 0.3 mg/mL in PB) for 10 minutes. Hydrogen peroxide (0.003%) was added and the sections were incubated for 1 hour in the dark. They were then rinsed and osmicated (1% osmium tetroxide and 1.5% potassium ferrocyanide for 1 hour) before being dehydrated in graded acetone solutions, block stained in uranyl acetate, and flat-embedded in Durcupan, as described previously.^62^ Ultrathin sections (silver interference colour) were cut with an Ultracut S ultramicrotome, mounted on formvar-coated slot grids, and viewed with a Philips CM100 electron microscope equipped with a digital camera.

### 2.8. Electrophysiology

Electrophysiological recordings from GRPR cells were performed on spinal cord slices or on isolated spinal cord preparations that were obtained from 91 GRPR^CreERT2^;Ai9 mice and 5 GRPR^CreERT2^ mice (age range 5-14 weeks, mean 7.5 weeks), as described previously.^16^ The 5 GRPR^CreERT2^ mice had received intraspinal injections of AAV.flex.eGFP into the L3 and L5 segments on one side. After perfusion with ice-cold dissection solution, the spinal cord was isolated, and for experiments that involved dorsal root stimulation, the L3 and L4, or L4 and L5, dorsal roots were left attached to the cord. To prepare spinal cord slices, the lumbar region was embedded in low–melting point agar (∼3%; Thermo Fisher Scientific) and parasagittal (300-500 μm) or transverse (300-500 μm) slices were cut with a vibrating blade microtome (Microm HM 650V; Thermo Fisher Scientific or 7000smz-2; Campden Instruments). Slices were placed in *N*-methyl-d-glucamine (NMDG)-based recovery solution at 32°C for ∼15 minutes and then in a modified recording solution at room temperature for an additional 1 hour before being transferred to the recording chamber of an upright microscope where they were continually perfused with recording solution. For experiments involving isolated spinal cord preparations, a length of spinal cord containing the lumbar segments, along with much of the thoracic and sacral regions, was prepared by removing the pia mater. In some cases, the spinal cord was hemisected by cutting along the midline using a razor blade. These preparations, referred to as “whole cord” or “hemisected cord,” were placed into the recording chamber and pinned to the chamber wall, which was made from Sylgard. The recording chamber was placed on the microscope stage, and tissue was continually perfused with recording solution. The solutions used contained the following (in mM): Dissection: 3.0 KCl, 1.2 NaH_2_PO_4_, 0.5 CaCl_2_, 7.0 MgCl_2_, 26.0 NaHCO_3_, 15.0 glucose, and 251.6 sucrose; NMDG recovery: 93.0 NMDG, 2.5 KCl, 1.2 NaH_2_PO_4_, 0.5 CaCl_2_, 10.0 MgSO_4_, 30.0 NaHCO_3_, 25.0 glucose, 5.0 Na ascorbate, 2.0 thiourea, 3.0 Na pyruvate, and 20.0 HEPES; modified recording: 92.0 NaCl, 2.5 KCl, 1.2 NaH_2_PO_4_, 2.0 CaCl_2_, 2.0 MgSO_4_, 30.0 NaHCO_3_, 25 glucose, 5.0 Na ascorbate, 2.0 thiourea, 3.0 Na pyruvate, and 20.0 HEPES; recording: 125.8 NaCl, 3.0 KCl, 1.2 NaH_2_PO_4_, 2.4 CaCl_2_, 1.3 MgCl_2_, 26.0 NaHCO_3_, and 15 glucose. All solutions were bubbled with 95% O_2_ and 5% CO_2_.

Neurons were visualised using a fixed stage upright microscope (BX51; Olympus, Southend-on-Sea, UK) equipped with a 40× water immersion objective, infrared differential interference contrast (IR-DiC) illumination, and a CCD camera (QImaging Retiga Electro; Teledyne Photometrics, Birmingham, United Kingdom). In the case of whole or hemisected spinal cord preparations, cells were visualised using oblique IR illumination from an 860 nm LED (Opto SFH 4550; Osram, Munich, Germany).^59^ Fluorescently labelled cells were visualised using a 470 nm or 550 nm LED (pE-100; CoolLED, Andover, United Kingdom). Targeted whole-cell patch-clamp recordings were made from tdTomato-positive or GFP-positive cells in the SDH, using patch pipettes that had a typical resistance of 3 to 7 MΩ when filled with an intracellular solution containing the following (in mM): 130.0 K gluconate, 10.0 KCl, 2.0 MgCl_2_, 10.0 HEPES, 0.5 EGTA, 2.0 ATP-Na, 0.5 GTP-Na, and 0.2% Neurobiotin, pH adjusted to 7.3 with 1.0 M KOH. In some cases, the intracellular solution contained the following (in mM); 120.0 Cs methanesulphonate, 10.0 Na methanesulphonate, 1.0 CaCl_2_, 10.0 HEPES, 10.0 EGTA, 2.0 ATP-Mg, 5.0 QX-314-Cl, and 0.2% Neurobiotin, pH adjusted to 7.3 with 1.0 M CsOH. Data were recorded and acquired with a Multiclamp 700B amplifier and pClamp 10 software (both Molecular Devices, Wokingham, United Kingdom) and were filtered at 4 kHz and digitised at 10 kHz.

After stable whole-cell configuration was achieved, the cells were voltage clamped at −60 mV, and a series of 100-millisecond voltage steps from −70 to −50 mV (2.5 mV increments) was delivered to determine the current–voltage relationship, which was used to calculate resting membrane potential. Cells with a resting membrane potential less negative than −30 mV were excluded from all analysis. Input resistance was calculated from a series of five -5mV steps of 1-second duration from a holding potential of −60 mV.

Action potential firing patterns were assessed in current clamp mode in response to 1-second depolarising current steps of increasing amplitude (5 pA increments) from a membrane potential of around −60 mV. Firing patterns were classified on the basis of previously published criteria^16,19,20,22,52,55,67^ as follows: tonic firing if they exhibited a continuous action potential discharge throughout the step, transient if the action potential discharge occurred only during the early part of the step, delayed if there was a clear delay between the start of the depolarising step and the first action potential, single spike if only 1 or 2 action potentials occurred at the onset of the step, and reluctant if current injection did not evoke action potential firing. Action potential properties for each neuron were determined from the first action potential that occurred at rheobase.

Subthreshold voltage-activated currents were investigated by holding cells at −60 mV before stepping to −90 mV for 1 second and then to −40 mV for 200 milliseconds, with automated leak subtraction to remove capacitive and leak currents.^21,55^ This protocol enables identification of 2 types of transient outward current and 2 types of inward current. The outward currents that occur during the depolarising step (−90 to −40 mV) are consistent with A-type potassium currents (I_A_) and can be identified as rapid (I_Ar_) or slow (I_As_) on the basis of their kinetics. A transient inward current observed during the depolarising step is considered to reflect the low-threshold “T-type” calcium current (I_Ca,T_). A slow inward current during the hyperpolarisation step (−60 to −90 mV) is classified as the hyperpolarisation-activated (I_h_) current. The amplitude of I_Ar_ was measured as the peak of the transient outward current. The amplitude of the I_h_ current was measured during the final 200 milliseconds of the hyperpolarising step, and inward currents were classified as I_h_ if the amplitude was greater than −5 pA.

Primary afferent input to GRPR cells was assessed by recording evoked excitatory postsynaptic currents (eEPSCs) in spinal cord slices or in the whole or hemisected spinal cord preparation with dorsal roots attached, in response to dorsal root stimulation, as described previously.^31,63^ Cells were voltage clamped at −70 mV, and the dorsal root stimulated through a suction electrode connected to an ISO-Flex stimulus isolator (AMPI, Jerusalem, Israel). In many cases, 2 dorsal roots were attached, either L3 and L4, or L4 and L5, with each being stimulated independently using a separate suction electrode and stimulus isolator. To characterise the type of primary afferent input to cells, the dorsal root was stimulated 3 times at low frequency (0.05 Hz, 0.1 ms duration), using the following intensities to activate Aβ, Aδ, and C fibres: 25 µA for Aβ, 100 µA for Aδ, and 1 mA for C fibres.^31^ Primary afferent input was characterised as monosynaptic or polysynaptic by stimulating the dorsal roots 20 times using the following intensities and frequencies; Aβ: 25 µA, 20 Hz; Aδ: 100 µA, 2 Hz; and C: 1 mA, 1 Hz. Fibre responses were considered monosynaptic if there was an absence of response failures and the response latency varied by ≤2 ms, whereas C-fibre responses were classified as monosynaptic if there was an absence of failures, regardless of whether the response latency was variable.^43^ The estimated conduction velocity of monosynaptic responses was calculated using the response latency, measured as the time between the stimulus artefact and the onset of the eEPSC, and the length of the stimulated dorsal root, measured as the distance between the suction electrode and the dorsal root entry zone.

Excitatory synaptic input to GRPR neurons was assessed by voltage-clamping cells at −70 mV and recording spontaneous EPSCs (sEPSCs) and miniature EPSCs (mEPSCs); the latter in the presence of tetrodotoxin (TTX) (0.5 µM), bicuculline (10 µM), and strychnine (5 µM). The functional expression of TRP channels on the afferents providing synaptic input was investigated by recording mEPSCs before and during the bath application of the TRPV1 agonist capsaicin (2 µM) or the TRPM8 agonist WS-12 (1 µM). In the case of WS-12 application, the temperature of the bath was raised to 32°C using an inline heating system (Scientifica Ltd, Uckfield, United Kingdom).^31^ These data were analysed using Mini Analysis (Synaptosoft). The events were automatically detected by the software and were then rejected or accepted after visual examination. Neurons were considered to receive input from capsaicin-sensitive or WS-12-sensitive afferents if application of the agonist resulted in a significant leftward shift in the distribution of interevent intervals, indicating an increase in frequency, or nonresponsive if this threshold was not reached.

The response of GRPR cells to a number of pharmacological agents was investigated by voltage-clamping cells at −50 mV and bath applying one of the following, in the presence of TTX (0.5 µM), bicuculline (10 µM), and strychnine (5 µM): 5-HT (10 µM), norepinephrine (NE) (20 µM), the µ-opioid receptor (MOR) agonist DAMGO (3 µM), the k-opioid (KOR) agonists ICI 199,441 (100 nM) or U69593 (1 µM), or the δ-opioid (DOR) agonist [D-Ala^2^]-deltorphin II (1 µM). Cells were considered responsive if drug application resulted in a clear slow outward current that was greater than 5 pA. Responses of GRPR cells to GRP were similarly assessed by voltage-clamping cells at −50 mV and applying GRP (2 µM), with cells being considered responsive if GRP application resulted in a clear slow inward current that was greater than −5 pA. TTX, bicuculline, and strychnine were not included in the bath during GRP application to enable the study of synaptic connectivity between GRPR cells, by comparing sEPSCs recorded before and during the application of GRP.

All chemicals were obtained from Sigma, except sucrose, glucose, NaH_2_PO_4_ (Thermo Fisher Scientific), NaCl, KCl, HEPES (VWR, Lutterworth, United Kingdom), TTX, QX-314-Cl (Alomone, Jerusalem, Israel), bicuculline, DAMGO, ICI 199,441, WS-12 (Tocris, Abingdon, United Kingdom), GRP (Cambridge Bioscience, Cambridge, United Kingdom), NE (Abcam, Cambridge, United Kingdom), and Neurobiotin (Vector Laboratories, Peterborough, United Kingdom).

### 2.9. Phosphorylation of extracellular signal-regulated kinases after noxious or pruritic stimuli

Phosphorylated ERK (pERK) labelling was examined in 16 GRPR^CreERT2^;Ai9 mice that had received a noxious, pruritic, or control stimulus applied to 1 hind limb under urethane anaesthesia (50-60 mg, i.p). Three of these mice had the left hind limb immersed in water at 52°C for 15 seconds (noxious heat), and 4 mice had 5 different regions on the skin of the left calf pinched for 5 seconds each (noxious mechanical). These animals were perfused with fixative 5 minutes later. Three mice received one of the following intradermal injections into the left calf, which had been shaved the day before: histamine (100 μg in PBS), chloroquine (100 μg in PBS), or PBS. In all cases, the injection volume was 10 μL and success of the intradermal injections was verified by the presence of a small bleb in the skin. These animals survived for 30 minutes before perfusion fixation because we have previously shown that intradermal injections of vehicle result in pERK labelling if mice are allowed to survive for only 5 minutes after the stimulus, presumably due to the noxious mechanical stimulus resulting from intradermal injection.^6^ However, if mice survive 30 minutes, pERK is seen in pruritogen-injected, but not vehicle-injected, animals, reflecting prolonged activation by pruritogens. Some of the animals used for the pERK experiments had been included in a previous study that investigated expression of neuropeptide FF in activated neurons.^23^

Transverse sections from the somatotopically appropriate segments of these animals (L3 for pruritogen or vehicle injection, or pinch and L4 for noxious heat) were reacted with antibodies against pERK and mCherry (chicken antibody). GRPR cells were initially identified in confocal scans with the pERK channel hidden. The proportion of GRPR cells that were pERK-positive was then determined.

### 2.10. Chemogenetic activation and behavioural testing

Experiments to test the effect of activating GRPR neurons were performed on 6 GRPR^CreERT2^ mice (3 homozygous female and 3 hemizygous male) that had received intraspinal injections of AAV.flex.hM3Dq-mCherry targeted on the L3, L4, and L5 segments on the right side. All mice used in this part of the study had undergone handling and acclimatisation to the testing chambers before the start of testing. Behavioural tests and video recording of behaviour were performed 2 weeks after the intraspinal injections and 1 week after tamoxifen treatment, using a crossover design in which each mouse was injected intraperitoneally with 0.2 mg/kg clozapine N-oxide (CNO, the ligand for hM3Dq) on one day and vehicle on another day (assigned in a random order). All testing and analyses were performed by the same experimenter, who was blind to the treatment type.

On test days, mice were placed into the video recording enclosures (consisting of 10 × 10 × 10-cm plexiglass chambers surrounded by 45°-angled mirrors) immediately after CNO or vehicle injection, and behaviour was recorded for 30 minutes using a Sony FDR-AX33 video camera. This was analysed offline using the free open-source BORIS event-logging software.^18^ Four separate behaviours were logged whenever they occurred within the relevant dermatomes (L3-5, covering the hind paw, hind leg, rump, and base of the tail) on the side ipsilateral to spinal injections: (1) biting of the skin, (2) licking of the hair and/or skin, (3) lifting and/or guarding of the paw, and (4) rapid shaking of the foot. Biting, licking, together with lifting and/or guarding were logged as state events with a defined duration per bout or episode, whereas paw shaking was logged as a point event. Because mouse movements can be extremely rapid, all behaviours were reviewed at 0.2× playback speed. Licking and biting due to normal grooming behaviour were included in the analyses if they occurred within the ipsilateral dermatome areas (as described above) because biting and licking as part of normal grooming could not be distinguished from those resulting from chemogenetic activation of the GRPR cells.

After these video recording periods, mice were transferred to the von Frey or Hargreaves testing apparatus and allowed to acclimatise for at least 30 minutes before testing began. Mechanical sensitivity was tested by application of a set of von Frey filaments with logarithmically increasing stiffness (starting at 0.4 g) to the glabrous skin of the ipsilateral hind paw through mesh flooring, as described previously.^29,47^ The 50% withdrawal threshold was determined by the up–down method.^12^ Heat sensitivity was tested with a Hargreaves apparatus (IITC, Woodland Hills, CA) consisting of a ∼16-mm thick glass plate warmed to 25°C, through which a radiant heat source (set to 25% active intensity) was targeted to the glabrous skin of the ipsilateral hind paw. The time taken to withdraw the paw was measured, and the average withdrawal latency across 5 trials was determined for each animal. A cutoff threshold of 25 seconds was used to prevent potential tissue damage. All behavioural testing was completed within 2.5 hours after administration of CNO or vehicle.

Once all behavioural testing was completed, the mice were fixed by perfusion and the location and extent of the injection sites were assessed by immunostaining spinal cord sections with chicken anti-mCherry antibody.

### 2.11. Antibody characterisation

The mCherry and GFP antibodies were raised against the full-length recombinant proteins, whereas the teal fluorescent protein (TFP) and tagRFP antibodies were raised against the corresponding purified proteins. In all cases, their specificity is demonstrated by the lack of immunostaining in tissue that lacks the corresponding fluorescent protein. Staining with the pro-NPFF antibody is completely abolished by preincubation with the antigen.^23^ Specificity of the pro-CCK antibody has been demonstrated by showing that it stains the same cells as those detected by fluorescence in situ hybridisation with a probe against *Cck* mRNA in the mouse cortex and hippocampus.^8^ Staining with the rat neurotensin is identical to that seen with a well-characterised rabbit anti-neurotensin antibody and is blocked by preincubation with neurotensin.^50^ The PPTB antibody recognises PPTB, but not PPTA, neurokinin B, or substance P, on dot blots, and staining is blocked by preincubation with the immunising peptide.^32^ The PKCγ antibody recognises a band of the appropriate molecular weight on western blots from wild-type, but not from PKCγ^−/−^, mice.^68^ The antibody against pERK detects p44 and p42 MAP kinase (Erk1 and Erk2) when these are phosphorylated either individually or dually at Thr202 and Tyr204 of Erk1 or Thr185 and Tyr187 of Erk2. It does not cross-react with nonphosphorylated Erk1/2. The Pax2 antibody recognises bands of the appropriate molecular weight on western blots of mouse embryonic kidney.^17^ The guinea pig NeuN antibody stains identical cells to a well-characterised mouse monoclonal NeuN antibody.^16^

### 2.12. Statistics

Recorded neurons were classified as responsive to capsaicin, WS-12, or GRP by comparing the cumulative probability distribution of mEPSC or sEPSC interevent intervals with a 2-sample Kolmogorov–Smirnov test. Changes in EPSC frequency between sEPSCs and mEPCSs, recorded in the same cell, and between baseline mEPSCs or sEPSCs and those recorded during capsaicin, WS-12, or GRP application were compared using Wilcoxon signed-rank tests. Mann–Whitney *U* tests were used to investigate differences between electrophysiological data that were recorded in female and male tissue. Electrophysiological properties of GRPR cells were compared with those previously recorded in GRP and SP cells^16^ by using Kruskal–Wallis multiple comparison tests, followed by Dunn multiple comparison tests. Normality of behavioural data was assessed using the Kolmogorov–Smirnov test; data that passed the normality test were analysed using paired t-tests, whereas nonnormally distributed data were analysed using Wilcoxon matched-pairs signed-rank tests. Data are expressed as mean ± SD, and *P* values of less than 0.05 were considered significant. Statistical tests were performed in Prism version 9 (GraphPad Software, San Diego, CA).

### 2.13. Data Availability

Data can be accessed from an open repository at the following link: http://dx.doi.org/10.5525/gla.researchdata.1285.

## 3. Results

### 3.1. Characterisation of gastrin-releasing peptide receptor cells identified in the GRPR^CreERT2^ mouse

**Figure 1.**
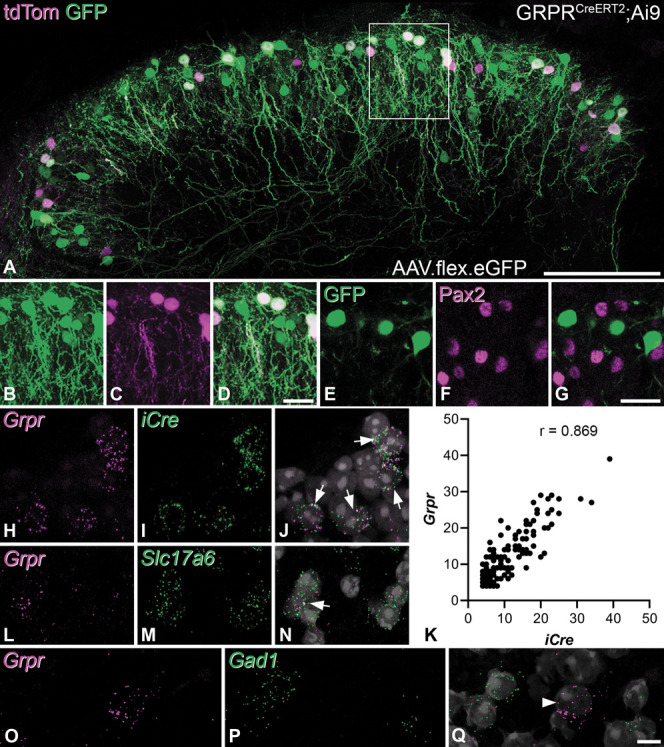
Characterisation of Cre-expressing cells in the GRPR^CreERT2^ mouse. (A) Intraspinal injection of AAV.flex.eGFP in a GRPR^CreERT2^;Ai9 mouse results in both GFP and tdTomato expression in cells with Cre. The GFP-expressing cells are more numerous, and virtually, all of these are also tdTomato (tdTom)-positive. Cells are concentrated in laminae I and IIo, although a few cells are present in laminae IIi-III. Note the predominantly ventral orientation of dendrites that originate from these cells. (B–D) Part of the superficial dorsal horn, corresponding to the box in (A), scanned to reveal GFP, tdTom, and both fluorescent proteins, respectively, shows that the GFP cells are more numerous. (E–G) Part of a section from a GRPR^CreERT2^ mouse that had received an intraspinal injection of AAV.flex.eGFP. The section has been stained for GFP and Pax2 and shows lack of Pax2 immunoreactivity in GFP-positive cells. (H,I) Part of the superficial dorsal horn from a GRPR^CreERT2^ mouse reacted for RNAscope in situ hybridisation with probes for *Grpr* and *iCre*, respectively. (J) A merged image is shown, together with nuclear staining. Four cells in this field are labelled with both probes. (K) Correlation between the number of transcripts for the 2 probes within 162 *Grpr-*positive and *iCre*-positive cells obtained from 2 mice. (L–N) and (O–Q) show sections from a wild-type mouse reacted with probes for *Grpr* and either *Slc17a6* (VGLUT2) or *Gad1*. In each of the RNAscope-merged images, arrows point to *Grpr+* cells that are also positive for *iCre* or *Slc17a6*, whereas the arrowhead indicates a *Grpr* cell that is negative for *Gad1*. (A–D) and (E–G) are projections of 31 and 4 confocal optical sections, respectively, at 1 μm z-separation. (H–J) and (L–Q) are projections from the full thickness of the cryostat sections. Scale bars (A): 100 μm, (B–D): 20 μm, (E–G): 20 μm, (H–J), and (L–Q): 10 μm. GRPR, gastrin-releasing peptide receptor; GFP, green fluorescent protein.

In experiments in which AAV.flex.eGFP was injected into the spinal cord of GRPR^CreERT2^;Ai9 mice, both tdTomato and GFP were present in many neurons in the SDH, and the pattern of GFP labelling was very similar with both AAV2 and AAV8 serotypes. The laminar distribution of GFP+ and tdTom+ cells was the same, with the majority being located in laminae I and IIo, as reported previously^41^ (Figs. 1A–D). However, we found that the GFP+ cells consistently outnumbered those with tdTom, and we therefore quantified this in 3 mice. Using the optical disector method on 2 sections per animal, we counted a mean of 44 GFP+ cells (range 31-54) within the injection site in each animal and found that 38.4% (36.2%-41.9%) of these also contained tdTomato. Conversely, all tdTomato+ cells in this sample were GFP+. This suggests that recombination efficiency is higher with intraspinal AAV injection than that in the Ai9 cross. We therefore quantified the proportion of all neurons in laminae I-II that were GFP+ in the L3 or L4 segments of homozygous GRPR^CreERT2^ mice that had received intraspinal injections of AAV.flex.eGFP into these segments by examining 2 transverse sections from each of 3 mice. We identified a mean of 400 neurons (range 359-455, identified by the presence of NeuN) in laminae I-II in these animals and found that 10.9% (9.9%-11.9%) of them were GFP+. In sections from 2 of these mice, we also immunostained for Pax2, which is expressed in inhibitory interneurons^14,35^ (Figs. 1E–G). We found that none of the 77 GFP+ neurons identified (38 and 39 in the 2 mice) were Pax2-immunoreactive, suggesting that GFP expression was restricted to excitatory neurons. Because we have previously reported that 74.2% of the neurons in laminae I-II of the mouse dorsal horn are excitatory,^48^ we estimate that the GFP+ cells account for 14.7% of the excitatory neurons in this region.

To confirm that expression of Cre was restricted to neurons with *Grpr* mRNA, we performed fluorescence in situ hybridisation with RNAscope (Figs. 1H–J). We analysed sections from 2 homozygous GRPR^CreERT2^ mice and identified 100 (range 100-101) cells with *Grpr* mRNA in laminae I-II in each case. Of these cells 81% also contained mRNA for *iCre* (at 4 or more transcripts per cell). In addition, 92 (90-95) *iCre* cells were identified and 88% of these were classed as *Grpr* positive. When cells defined as single labelled by this criterion were analysed, it was found that 38 of the 39 “*Grpr*-only” cells had at least 1 transcript for *iCre*, whereas 21 of 23 “*iCre*-only” cells had at least 1 transcript for *Grpr*. The number of individual transcripts for the 2 mRNAs showed a strong positive correlation (Pearson correlation = 0.869, *P* < 0.001) (Fig. 1K). These findings show that expression of Cre in this mouse line reliably recapitulates that of GRPR at the mRNA level.

Gastrin-releasing peptide receptor–expressing cells are believed to be excitatory interneurons,^64^ and this was recently confirmed by Sheahan et al.,^54^ who showed that 98.5% of SDH neurons with Grpr mRNA were also positive for Slc17a6 mRNA (VGLUT2 message). We determined the proportion of excitatory neurons in laminae I-II that possess Grpr mRNA in sections from 4 wild-type mice that had been reacted to reveal Grpr and Slc17a6 (VGLUT2) mRNAs (Figs. 1L–N). We identified 266 to 358 (mean 311) cells with Slc17a6 mRNA in these sections. Consistent with the findings of Sheahan et al., we found that all Grpr-positive cells (defined by the presence of at least 4 transcripts for Grpr mRNA) were Slc17a6-positive, and that these accounted for 17.2% (14.0%-20.4%) of the Slc17a6 cells. To exclude the possibility that some GRPR-expressing cells were inhibitory, we examined sections that had been reacted for Grpr and Gad1 mRNAs (Figs. 1O–Q). We identified a mean of 45 (range 43-48) Grpr-positive cells in sections from 3 mice and found that none of them contained Gad1 mRNA.

Together, these results suggest that cells with Grpr mRNA in the SDH are all glutamatergic, and that they account for 15% to 17% of the excitatory neurons in this region. Previous studies have shown that GRPR is not expressed by projection neurons in the SDH,^41,64^ and so these cells are presumably all excitatory interneurons.

### 3.2. Relation of gastrin-releasing peptide receptor cells to other excitatory interneuron populations

**Figure 2.**
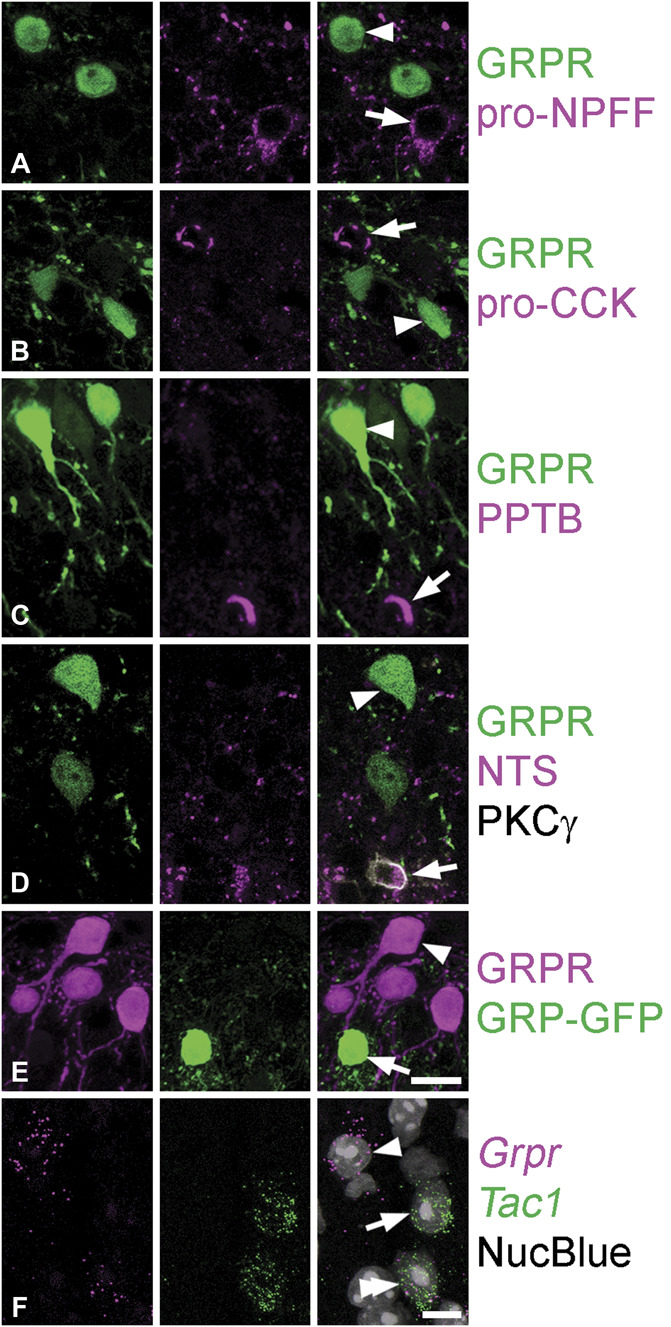
Relation of GRPR cells to other neurochemical populations. (A–D) Sections from GRPR^CreERT2^ mice that had received intraspinal injections of AAV.flex.eGFP. In each case, sections have been immunoreacted to reveal GFP (green), together with different peptides or propeptides (magenta): pro-NPFF, pro-CCK, preprotachykinin B (PPTB), and neurotensin (NTS). In (D), the section was also immunostained for PKCγ (grey), which outlines most neurotensin-containing cell bodies. In each case, there is no overlap between the GFP cells (one of which is indicated with an arrowhead) and cells expressing the (pro)peptides (one of which is marked with an arrow). (E) Part of a section from a GRPR^CreERT2^;Ai9;GRP::GFP mouse stained to reveal GRP (green) and tdTomato (magenta). Several GRPR cells (one of which is marked with an arrowhead) and a single GRP–GFP cell (arrow) are visible. (F) RNAscope in situ hybridisation on a section from a wild-type mouse reacted to reveal mRNAs for *Grpr* (magenta) and *Tac1* (green). Cells are revealed with NucBlue. The arrowhead and arrow indicate cells with only *Grpr* or *Tac1* mRNAs, respectively, whereas the double arrowhead shows a cell with both types of mRNA. (A and D) are single confocal optical sections, whereas (B, C, and E) are projections of 5, 6, and 7 confocal optical sections, respectively, at 1 μm z-separation. (F) is a projection from the full thickness of the cryostat section. Scale bars (A–E): 10 μm and (F): 10 μm. GRPR, gastrin-releasing peptide receptor; GFP, green fluorescent protein.

We then tested whether the GRPR cells overlapped with any of the excitatory interneuron populations that we have previously identified in the SDH.^23^ We used immunohistochemistry with antibodies that can be detected in the cell bodies of 4 of these populations (those defined by expression of NPFF, CCK, NKB, and neurotensin) (Figs. 2A–D). Scans were obtained from the full thickness of 2 to 3 sections each from GRPR^CreERT2^ mice that had received intraspinal injections of AAV.flex.eGFP, and we identified an average of 162 (98-199) GFP-positive cells per animal with each antibody combination. We found no overlap between PPTB (which is present in NKB-expressing cells) and GFP and only minimal overlap for the other 3 antibodies: 0.2% of GFP cells were pro-NPFF–immunoreactive or pro-CCK–immunoreactive, whereas 0.3% were neurotensin-immunoreactive. The GRP::GFP mouse from the GENSAT project reveals a specific subpopulation of GRP-expressing neurons,^7^ and we tested whether these overlapped with GRPR cells in the GRPR^CreERT2^;Ai9;GRP::GFP cross. We identified a mean of 100 tdTom+ cells in 3 mice and found that none of them were GFP+ (Fig. 2E). The lack of overlap between GRP–GFP and GRPR cells is consistent with the findings of Albisetti et al.,^1^ who reported no overlap between *Grp* and *Grpr* mRNAs in dorsal horn neurons.

We used in situ hybridisation with RNAscope to investigate possible overlap between GRPR and SP populations. This was performed on the sections reacted with probes for *Grpr* and *Tac1* that had been obtained from 3 wild-type mice (Fig. 2F). Of the 102 (80-121) *Grpr*+ cells identified, we found that 36% (35%-38%) were also positive for *Tac1* mRNA. In the converse analysis, we identified a mean of 153 (113-199) *Tac1*+ cells per animal and found that 25% (23%-26%) of these were *Grpr*+.

These findings indicate that the population of excitatory interneurons with *Grpr* mRNA is largely separate from the populations defined by expression of CCK, NPFF, neurotensin, NKB, or GFP in the GRP::GFP mouse. However, it shows a moderate degree of overlap with cells that express SP, with around one-third of GRPR cells having *Tac1* mRNA.

### 3.3. Somatodendritic morphology of gastrin-releasing peptide receptor cells

**Figure 3.**
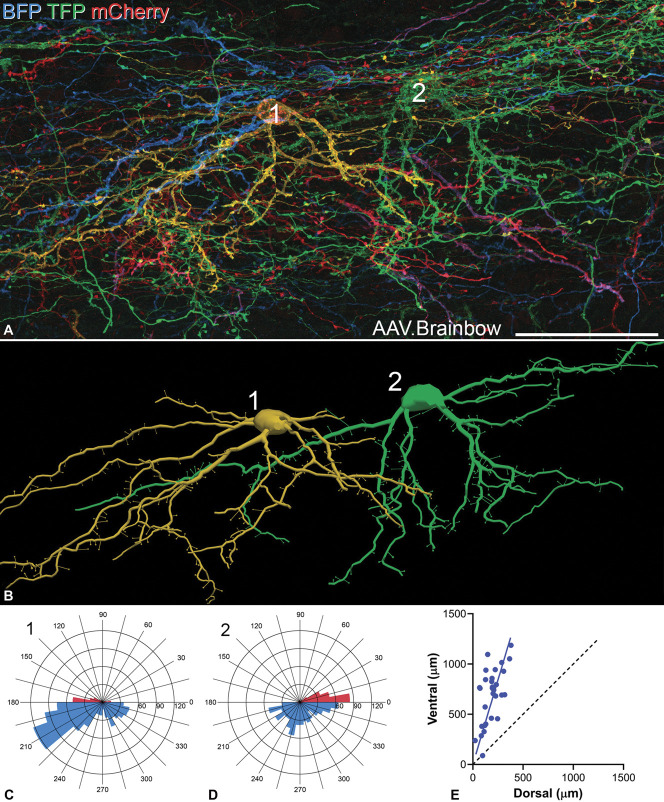
Somatodendritic morphology of GRPR cells as shown with the AAV Brainbow technique. (A) Part of a sagittal section from a GRPR^CreERT2^ mouse injected intraspinally with the Brainbow AAVs. The section has been scanned to reveal tagBFP (blue), TFP (green), and mCherry (red). Processes from numerous cells, each with a different colour, are visible in the section. Two cells that were reconstructed and included in the analysis are marked. (B) Neurolucida reconstructions of the 2 cells. Positions of dendritic spines are shown on these drawings, although the sizes of the corresponding symbols do not represent the actual sizes of spine heads. (C, D) Polar histograms for the 2 cells. The dorsally directed dendrites are shown in red and the ventrally directed dendrites in blue. (E) A plot of the ratio of ventral to dorsal dendritic lengths obtained from polar histograms from the 30 GRPR cells analysed in this study. The blue line shows the mean ratio for these cells, whereas the black dashed line corresponds to a ratio of 1. The image in (A) was generated from 109 confocal optical sections at 0.5 μm z-separation. Scale bar A = 50 μm. GRPR, gastrin-releasing peptide receptor; GFP, green fluorescent protein.

A recent report identified GRPR cells, revealed in a GRPR::GFP transgenic line from GENSAT, as having “vertical-type” morphology.^34^ Consistent with this, we noted that the GRPR cells seen either in the Ai9 cross or after injection of AAV.flex.eGFP had prominent ventrally directed dendrites (Fig. 1), which are characteristic of vertical cells.^22,67^ We therefore examined the somatodendritic morphology of these cells by using a viral Brainbow strategy.^11,16^ We reconstructed 30 neurons and found that all of these resembled the vertical cells described in numerous previous studies.^22,28,37,39,51,66,67^ All of the cells had dendritic spines, and the mean density was 15.9 ± 5.1 spines per 100 μm of dendritic length. The cell bodies of these neurons were located between 10 and 37 μm (mean 25 μm) below the dorsal surface of the white matter. Five of them were less than 20 μm below this surface and are therefore likely to have been in lamina I,^19^ whereas the remainder were in lamina IIo. Representative examples are shown in Figures 3A and B. One defining characteristic of vertical cells is that the extent of their ventral dendrites is much greater than that of their dorsal dendrites. We therefore created polar histograms (Figs. 3C and D) for each cell. These represent the lengths of dendrite that lie within specific ranges of orientation when the dendritic tree is projected onto the plane of section. We measured the total lengths of dendrite that had a dorsal or ventral orientation for each cell and plotted these values (Fig. 3E). This revealed that the lengths of ventrally directed dendrites always exceeded those of dorsally directed dendrites.

### 3.4. Axonal projections of gastrin-releasing peptide receptor cells

**Figure 4.**
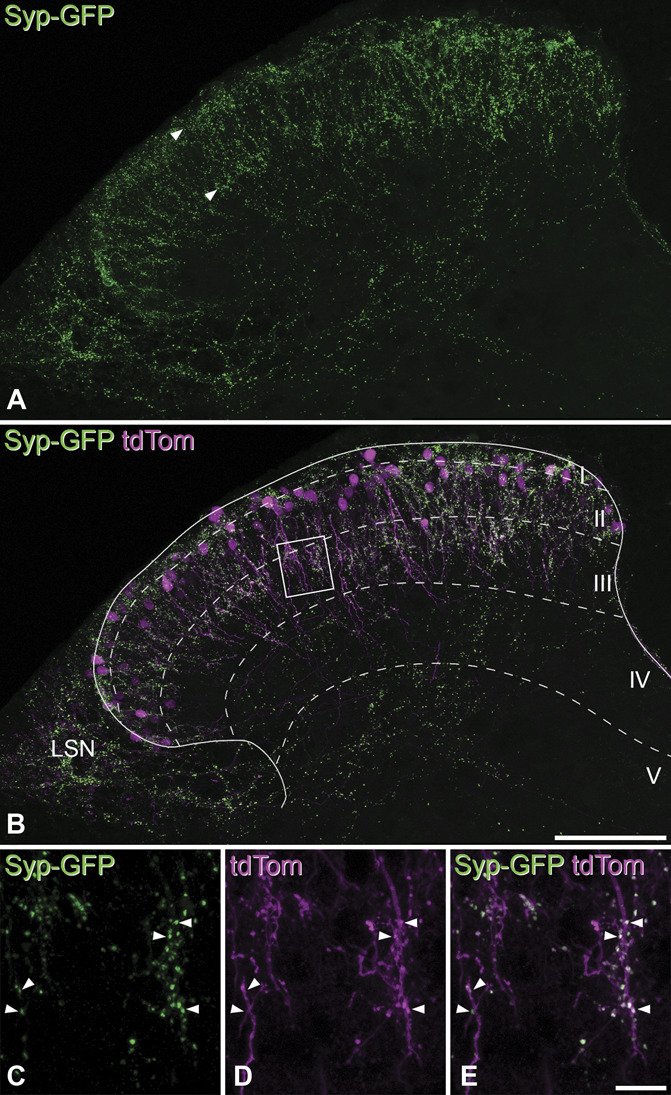
Axons of GRPR cells seen with AAVs coding for synaptophysin-GFP and tdTomato. (A, B) Immunostaining for GFP (A) and for both GFP and tdTomato (tdTom) (B) in a transverse section through the lumbar spinal cord from a GRPR^CreERT2^ mouse that had received injections of AAVs coding for Cre-dependent constructs expressing tdTomato and synaptophysin-GFP (Syp-GFP). GFP (green) is targeted to the axon terminals of GRPR cells, and these are present throughout laminae I and II, with relatively high concentrations in 2 bands (arrowheads): one corresponding to lamina I and the other the lamina II/III border. Axonal boutons are also present in the lateral spinal nucleus (LSN) and scattered through the deeper laminae of the dorsal horn and the lateral white matter. (C–E) A higher-magnification view of an area around the lamina II/III border, corresponding to the box in (B). Many of the GFP-labelled axonal boutons (some indicated with arrowheads) form contacts on the tdTom-labelled (magenta) dendrites of the GRPR cells. (A and B) and (C–E) are projections of 31 optical sections at 1 μm z-separation and 29 optical sections at 0.3 μm, respectively. Scale bars A and B = 100 μm and C–E = 10 μm. GRPR, gastrin-releasing peptide receptor; GFP, green fluorescent protein.

Axons of the GRPR cells could not be easily followed in Brainbow preparations, and we therefore used alternative approaches to investigate their distribution. We initially coinjected 2 AAVs that contained different Cre-dependent cassettes into spinal cords of GRPR^CreERT2^ mice. One of these coded for both tdTomato and a synaptophysin-GFP fusion protein (AAV.flex.tdTom_syp-eGFP), and the other encoded tdTomato (AAV.flex.tdTom) (Fig. 4). This combination was used because the first AAV generated relatively weak tdTomato expression. In transverse sections from this tissue, we observed a large number of GFP-labelled axonal boutons. These were particularly numerous in laminae I-II but were also seen in the lateral spinal nucleus and in deeper regions, particularly in the lateral part of lamina V and the adjacent white matter. Within the SDH, it was often possible to recognise 2 distinct bands of axonal labelling: one in lamina I and the outermost part of lamina II and one near the border between laminae II and III (arrowheads in Fig. 4A). Surprisingly, we found that many of the GFP-labelled axonal boutons were in contact with tdTom-labelled ventral dendrites of GRPR cells (Figs. 4C–E).

**Figure 5.**
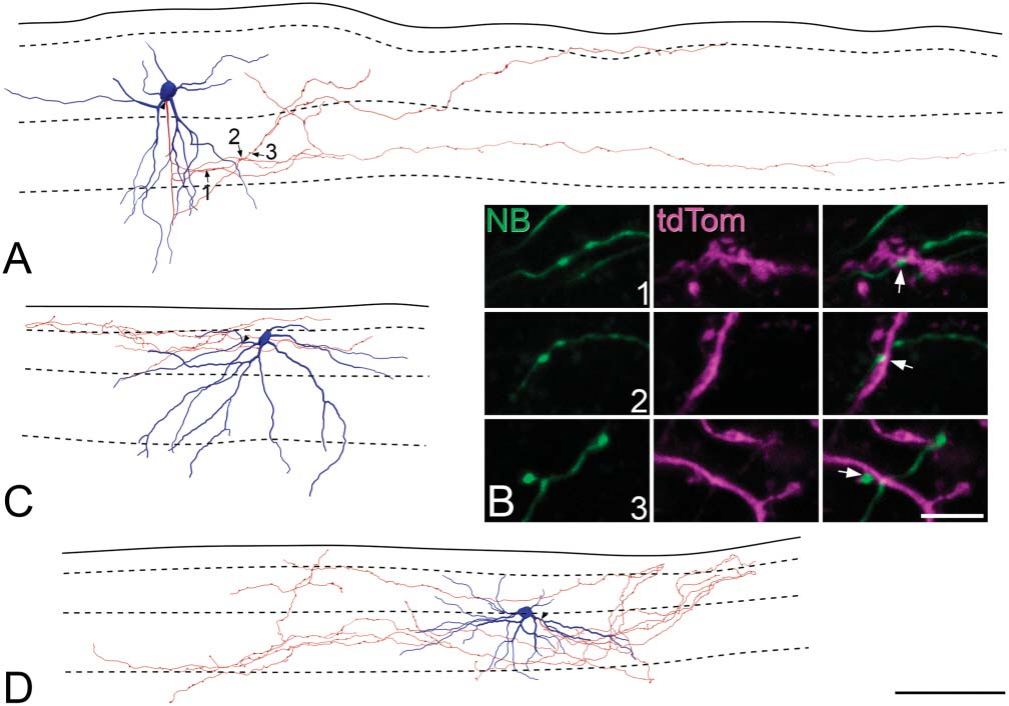
Axons of GRPR cells seen from reconstructions of patched neurons. (A, C, D) Typical examples of GRPR neurons reconstructed with Neurolucida after whole-cell recordings from tdTomato-positive cells in slices from GRPR^CreERT2^;Ai9 mice. Cell bodies and dendrites are shown in blue, and axons are in red. Note that dendritic spines have not been included in these drawings. The solid line shows the dorsal border of the grey matter, and the dashed lines show the approximate positions of the borders between laminae I, IIo, IIi, and III. Arrowheads indicate the origin of the axon from the soma (A) or a proximal dendrite (C, D). (B) Contacts formed by the axon of the cell illustrated in (A) (labelled with Neurobiotin, green) onto dendrites of other nearby tdTomato (tdTom, magenta) cells that were not patched are shown. Images in the top, middle, and bottom rows of (B) are projections of 7, 11, and 8 confocal scans at 0.3 μm z-separation, respectively. Scale bars A, C, and D = 100 μm and B = 5 μm. GRPR, gastrin-releasing peptide receptor.

We also reconstructed the axons of 13 Neurobiotin-filled GRPR cells that had undergone whole-cell recording (from 5 different animals), and examples are shown in Figure 5. Axons arose from either proximal portions of primary dendrites or cell bodies. The mean total length of axon that was reconstructed was 2162 µm ± 990 µm, with a range of 655 to 4411 µm. The axons generally remained in lamina II with an average of 50% of the reconstructed length in lamina IIo and 35% in lamina IIi. Some axons (11%) were present in lamina I or lamina III (4%). Because we had observed that axonal boutons of GRPR cells frequently contacted GRPR cell dendrites, we looked for contacts between the axons of Neurobiotin-filled patched cells and dendrites belonging to neighbouring tdTom-labelled cells that had not been patched. Many such contacts were evident, and examples are shown in Fig 5B.

**Figure 6.**
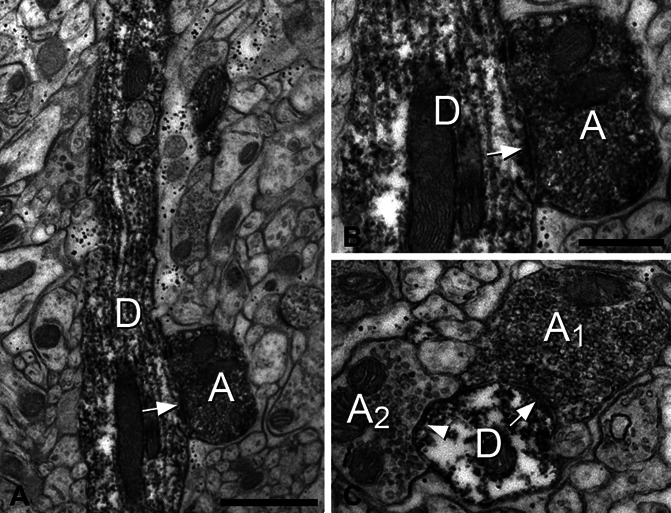
Electron microscopy of synapses between GRPR cells. Electron micrographs showing parts of lamina II from a GRPR^CreERT2^ mouse that had received a spinal injection of AAV.DIO.dAPEX2, resulting in cytoplasmic peroxidase expression, which is revealed with diaminobenzidine. (A) Part of a peroxidase-labelled dorsoventrally directed dendritic shaft D can be seen, together with a labelled axonal bouton A. An asymmetrical synapse is visible between them (arrow), and this is more clearly seen at higher magnification in (B). (C) A peroxidase-labelled dendrite shaft (cut in cross-section) receives an asymmetrical synapse (arrow) from a peroxidase-labelled axonal bouton (A_1_) and a symmetrical synapse (arrowhead) from an unlabelled axonal bouton (A_2_). Scale bars A = 1 μm and B and C = 0.5 μm. GRPR, gastrin-releasing peptide receptor.

Together, these findings suggest that axons of GRPR cells frequently synapse onto other GRPR cells. To confirm this, we used a recently developed viral strategy that depends on recombinase expression^69^ to deliver peroxidase to the GRPR cells. After a DAB reaction to reveal the peroxidase, we found numerous DAB-labelled profiles with the expected distribution in the SDH of the injected segments. These included cell bodies, dendrites, and axonal terminals, and the latter could readily be distinguished by the presence of densely packed synaptic vesicles. We found many examples of DAB-labelled axons forming asymmetrical synapses onto DAB-labelled dendrites (Fig. 6), confirming the prediction that the GRPR cells receive numerous synapses from other GRPR cells.

### 3.5. Electrophysiological properties of gastrin-releasing peptide receptor cells

With a few exceptions, described below, no sex differences were observed between cells recorded in female and male mice, and therefore, data have been pooled from cells recorded in both sexes.

**Figure 7.**
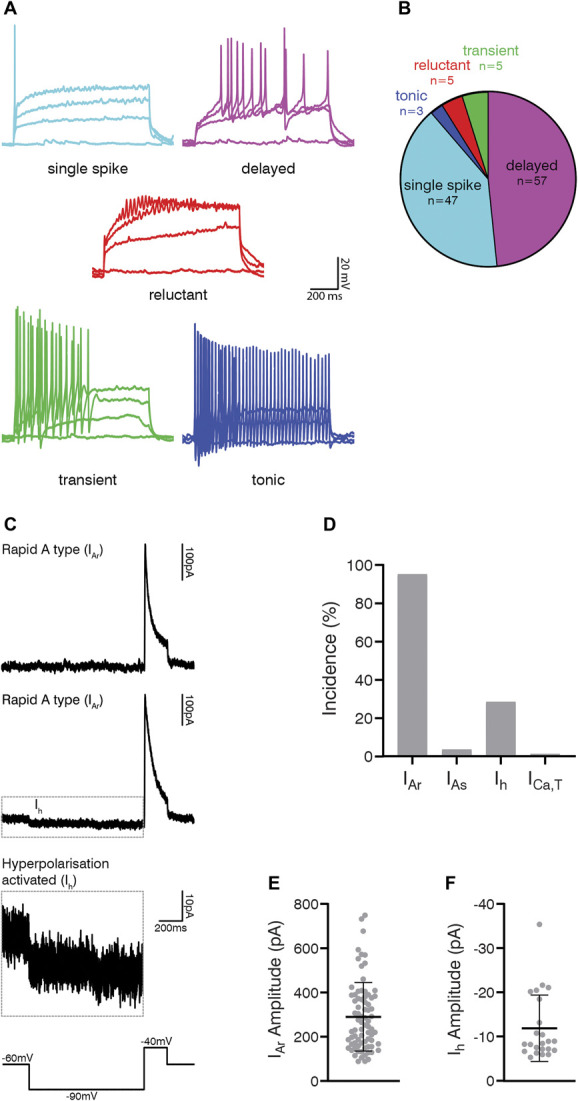
Action potential firing patterns and subthreshold voltage-activated currents in GRPR cells. (A) Examples of action potential firing patterns observed in GRPR cells in response to 1-second suprathreshold current injections. (B) Most GRPR cells exhibited delayed (57/117; 48.7%) or single-spike (47/117; 40.2%) firing patterns, with smaller proportions displaying transient (5/117; 4.3%), reluctant (5/117; 4.3%), or tonic (3/117; 2.6%) firing patterns. (C) Representative traces showing subthreshold voltage-activated currents in GRPR cells that were revealed using a voltage-step protocol that hyperpolarised cells from −60 to −90 mV for 1 second and then to −40 mV for 200 milliseconds (bottom trace). The responses to this protocol were classified as rapid (I_Ar_) or slow (I_As_) A-type potassium currents, hyperpolarisation-activated currents (I_h_), or low-threshold calcium currents (I_Ca,T_). Examples of I_Ar_ without (top trace) and with I_h_ (upper-middle trace) show an average of 5 traces. The example of I_h_ (dashed outline) is shown at a different y-axis scale (lower-middle trace). (D) Almost all GRPR cells exhibited I_Ar_ (77/81; 95.1%) and many had I_h_ (23/81; 28.4%), which was almost exclusively seen in addition to I_Ar_ (22/81; 27.2%). Few cells exhibited I_As_ (3/81; 3.7%) or I_Ca,T_ (1/81; 1.2%). The peak amplitude of I_Ar_ (E) was 289.8 ± 154.7 pA, and the amplitude of I_h_ (F), measured during the final 200 ms of the hyperpolarisation step, was −11.9 ± 7.5 pA. GRPR, gastrin-releasing peptide receptor.

Gastrin-releasing peptide receptor cells generally exhibited delayed (57/117, 48.7%) or single-spike (47/117, 40.2%) firing patterns in response to depolarising current steps. A small proportion of cells displayed transient (5/117, 4.3%), reluctant (5/117, 4.3%), or tonic (3/117, 2.6%) firing patterns (Figs. 7A and B). We noted that delayed firing was more commonly seen in cells recorded from female mice (48/89, 54%) than in those from male mice (9/28, 32%), although this difference was not statistically significant (Fisher exact probability test *P* = 0.053).

Membrane properties of the GRPR cells are provided in Table S1 (available as supplemental digital content at http://links.lww.com/PAIN/B638), and mean values are described in the following section. The resting membrane potential of the GRPR cells was −58.9 mV, their input resistance was 767.2 MΩ, and their capacitance was 9.88 pF. The rheobase current was 68.7 pA, with the following parameters measured from the first action potential at rheobase: the voltage threshold for evoking action potential firing (defined as the point where the rate of voltage rise exceeded 10 mV/ms) was −30.0 mV, the latency from the start of the depolarising step to the first action potential was 349.5 ms, base width was 1.4 ms, action potential height (measured as the difference between the voltage threshold and the action potential peak) was 45.2 mV, and after-hyperpolarisation was −25.9 mV. Two of these measures showed significant differences between female and male mice: latency to first action potential (female 407.2 ± 389.7 vs male 165.7 ± 273.0 ms, *P* = 0.024, Mann–Whitney) and action potential width (female 1.47 ± 0.37 vs male 1.30 ± 0.28 ms. *P* = 0.014, Mann–Whitney). The difference in latency presumably reflects the higher incidence of delayed firing neurons that were seen in tissue from females.

Almost all GRPR cells tested (77/81, 95.1%) displayed a rapid I_A_ current (I_Ar_), and many showed a hyperpolarisation-activated current (I_h_) (23/81, 28.4%), which in all but one cell was seen in conjunction with I_Ar_ (Figs. 7C and D, Table S1, available as supplemental digital content at http://links.lww.com/PAIN/B638). The peak amplitude of I_Ar_ was 289.7 pA (Fig. 7E), whereas I_h_ amplitude was −11.9 pA (Fig. 7F). Few cells exhibited slow I_A_ currents (I_As_) (3/81, 3.7%) or low-threshold Ca currents (I_Ca,T_) (1/81, 1.2%).

Comparison with our previous findings^15^ revealed that the GRPR cells had a significantly higher capacitance, lower input resistance, more negative resting potential, higher rheobase, and more negative action potential threshold than cells that expressed GFP in the GRP::GFP line, (Table S1, Fig. S1, available as supplemental digital content at http://links.lww.com/PAIN/B638). These results match the findings of Pagani et al.,^44^ who also compared these populations, and together indicate that the GRPR cells are larger and less excitable than the GRP–GFP cells. The GRPR cells also had a significantly lower action potential width and height, and a smaller after-hyperpolarisation, than the GRP-GFP cells. The values for the GRPR cells only differed significantly from those of SP-expressing cells only in having a higher capacitance and a more hyperpolarised resting membrane potential, together with a lower action potential latency and width. The greater similarity between these 2 populations presumably reflects, at least in part, the overlap in expression between GRPR and Tac1. For subthreshold currents, the GRPR cells resembled SP cells in having a high proportion with I_Ar_, and they also showed a significantly smaller I_h_ amplitude than the GRP–GFP cells (Table S1, Fig. S1, available as supplemental digital content at http://links.lww.com/PAIN/B638).

### 3.6. Primary afferent input to gastrin-releasing peptide receptor cells

**Figure 8.**
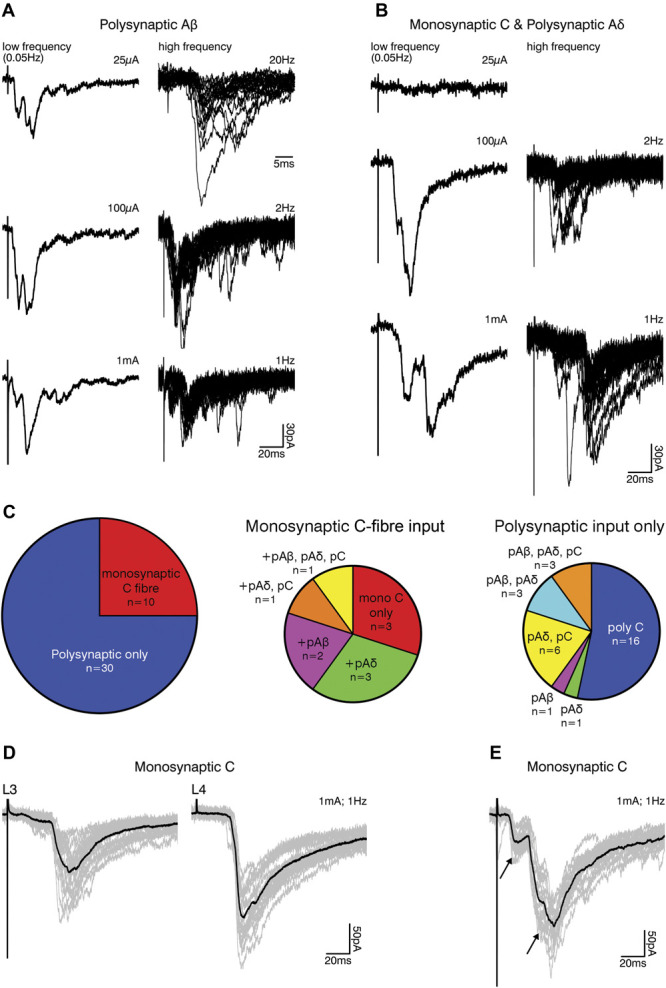
Primary afferent input to GRPR cells. (A, B) Examples of primary afferent input to GRPR cells classified as polysynaptic Aβ or monosynaptic C with polysynaptic Aδ in response to dorsal root stimulation are shown in (A) and (B), respectively. Left panels show evoked EPSCs in response to stimulation at low frequency (0.05 Hz) at intensities to activate Aβ (25 µA), Aδ (100 µA), and C (1 mA) fibres, each is an average of 3 traces. Right panels show eEPSCs resulting from high-frequency dorsal root stimulation (25 µA, 20 Hz; 100 µA, 2 Hz; and 1 mA, 1 Hz), each displays 20 superimposed traces. (C) A quarter of cells received monosynaptic C-fibre input (10/40; 25.0%, left chart), whereas the remainder received input that was classified as polysynaptic (30/40; 75.0%). In those cells that received monosynaptic C-fibre input (middle chart), this was mostly without additional input (3/10; 30.0%) or with input classified as polysynaptic Aδ (“pAδ”) (3/10; 30.0%) or polysynaptic Aβ (“pAβ”) (2/10; 20.0%). The remaining cells with monosynaptic C input additionally had polysynaptic Aδ with polysynaptic C (“pC”) (1/10; 10%) or polysynaptic input from Aβ, Aδ, and C fibres (1/10; 10%). In cells that only received input that was classified as polysynaptic (right chart), most of this input was from C fibres, either alone (16/30; 53.3%) or in addition to Aδ (6/30; 20.0%) or Aβ and Aδ (3/30; 10.0%) fibre input. Cells also received polysynaptic input that was classified as Aβ (1/30; 3.3%), Aδ (1/30; 3.3%), or Aβ and Aδ (3/30; 10.0%). Some cells were found to receive input from 2 separate dorsal roots (9/40; 22.5%). An example of a cell that received monosynaptic C-fibre input from both L3 and L4 dorsal roots is shown in (D). (E) In 1 cell, 2 separate monosynaptic C-fibre components could be identified after stimulation of a single (L5) dorsal root. In (D and E) 20 individual traces are shown in grey, with the average in black. eEPSCs, evoked excitatory postsynaptic currents; GRPR, gastrin-releasing peptide receptor.

Dorsal root stimulation was used to investigate primary afferent input to GRPR cells in spinal cord slices and in whole or hemisected spinal cords with attached dorsal roots. Electrical stimulation of dorsal roots resulted in eEPSCs in 40 of the 48 cells (83.3%) tested (representative traces shown in Figs. 8A and B). The 8 cells without eEPSCs were mostly recorded in spinal cord slice preparations (4 transverse slices and 1 parasagittal slice) and may have received input from axons that were severed during tissue slicing. These cells, along with 3 cells recorded in the whole-cord preparation that exhibited no eEPSCs, may also have received input from dorsal roots that were not stimulated. Of those cells that responded to dorsal root stimulation, most received input that was classified as polysynaptic (30/40, 75.0%). This was mostly polysynaptic C-fibre input but also included polysynaptic Aβ and polysynaptic Aδ input (Fig. 8C). The remaining cells (10/40, 25%) received monosynaptic C-fibre input, which was observed in the absence of other currents or with additional polysynaptic input(s). The estimated conduction velocity of the monosynaptic C-fibre input was 0.11 ± 0.02 m/second (n = 7), and the peak eEPSC amplitude was −157.2 ± 92.7 pA (n = 6). In those preparations where 2 dorsal roots were stimulated, 9 cells received input from both dorsal roots. This input was a mixture of monosynaptic C input (Fig. 8D) and/or polysynaptic input. In 1 cell, 2 clear monosynaptic C-fibre components could be observed during stimulation of a single dorsal root (Fig. 8E).

### 3.7. Excitatory input to gastrin-releasing peptide receptor cells

**Figure 9.**
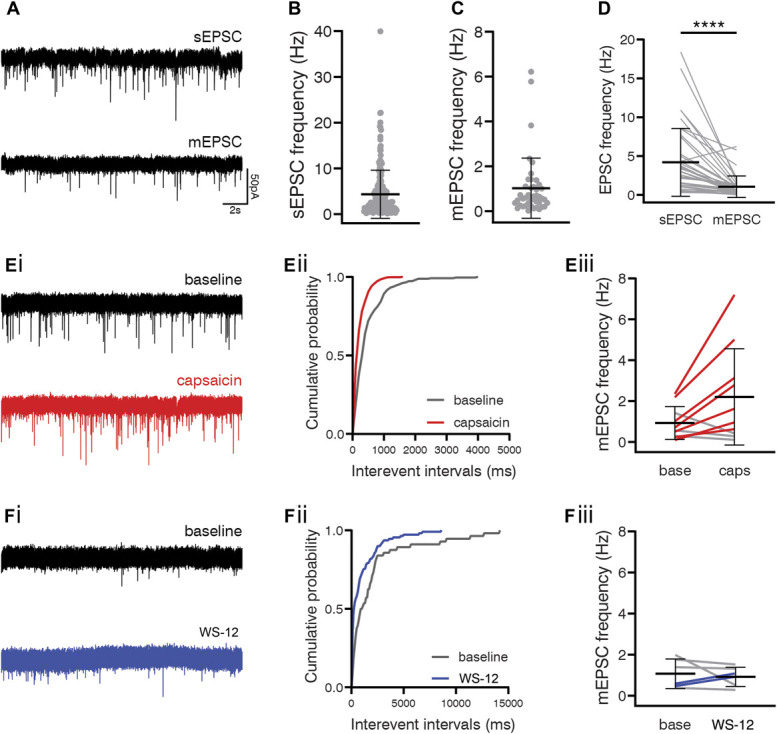
Excitatory synaptic input to GRPR cells. (A) Example traces of spontaneous (top) and miniature (bottom) EPSCs recorded in the same GRPR cell. The frequency of sEPSCs was 4.3 ± 5.3 Hz, n = 189 (B), and the frequency of mEPSCs was 1.0 ± 1.3 Hz, n = 42 (C). (D) In those cells where both sEPSCs and mEPSCs were recorded, the frequency of sEPSCs was significantly greater, 4.2 ± 4.4 vs 1.1 ± 1.4 Hz, n = 39, *P* < 0.0001, Wilcoxon signed-rank test. (E, F) The functional expression of TRP channels on primary afferent input to GRPR cells was assessed by recording mEPSCs in response to the application of agonists for TRPV1 (capsaicin; E) or TRPM8 (WS-12; F). Representative traces of mEPSCs recorded before (baseline; top) and during application of the TRP channel agonists (bottom) are shown in (Ei, Fi). Cumulative probability plots demonstrate a significant leftward shift in the distribution of mEPSC interevent intervals in response to the application of capsaicin (*P* < 0.00001, Kolmogorov–Smirnov 2-sample test; Eii) or WS-12 (*P* = 0.0003, Kolmogorov–Smirnov 2-sample test; Fii). A significant leftward shift in interevent intervals, signifying an increase in mEPSC frequency, was observed in 7 of 10 cells treated with capsaicin and 2 of 8 treated with WS-12. These agonists increased mEPSC frequency in those cells that responded as follows: capsaicin, 1.0 ± 0.9 Hz to 3.0 ± 2.4 Hz (Eiii) and WS-12, 0.5 ± 0.1 Hz to 1.0 ± 0.1 Hz (Fiii). Coloured lines in graphs represent cells that responded to the agonists, and grey lines represent cells that did not. EPSCs, excitatory postsynaptic currents; GRPR, gastrin-releasing peptide receptor.

Excitatory synaptic input to GRPR cells was assessed by recording sEPSCs and mEPSCs at a holding current of −70 mV (Fig. 9A). The EPSC frequency was 4.34 ± 5.26 (n = 189) and 1.02 ± 1.34 Hz (n = 42) for sEPSCs and mEPSCs, respectively (Figs. 9B and C). For 39 cells, we were able to compare sEPSCs and mEPSCs in the same cell. The sEPSC frequency in these cells was 4.20 ± 4.36 Hz, which was greater than the mEPSC frequency (1.06 ± 1.39 Hz), and this difference was highly significant (*P* < 0.0001, Wilcoxon signed-rank test, Fig. 9D). This finding suggests that GRPR cells receive excitatory synaptic input from neurons that were spontaneously firing action potentials in the slice. The GRPR cells had a higher sEPSC and mEPSC frequency than GRP–GFP cells but a lower mEPSC frequency than SP cells (Fig. S1, Table S1, available as supplemental digital content at http://links.lww.com/PAIN/B638).

Capsaicin caused a leftward shift in the distribution of mEPSC interevent intervals in 7 of 10 cells tested (Fig. 9Ei, Eii), resulting in an increase in frequency from 1.01 ± 0.91 to 3.04 ± 2.36 Hz in those capsaicin-sensitive cells (*P* = 0.016, Wilcoxon signed-rank test, Fig. 9Eiii). The TRPM8 agonist, WS-12, produced a leftward shift in the distribution of mEPSC interevent intervals in 2 of 6 cells (Fig. 9Fi, Fii), increasing mEPSC frequency in those cells from 0.53 ± 0.08 to 1.00 ± 0.11 Hz (Fig. 9Fiii). Because TRPV1 and TRPM8 channel expressions in the dorsal horn are believed to be restricted to primary afferents, these findings indicate that GRPR cells receive direct monosynaptic input from TRPV1-expressing primary afferents and to a lesser degree from TRPM8-expressing afferents.

### 3.8. Responses of gastrin-releasing peptide receptor cells to neuromodulators

**Figure 10.**
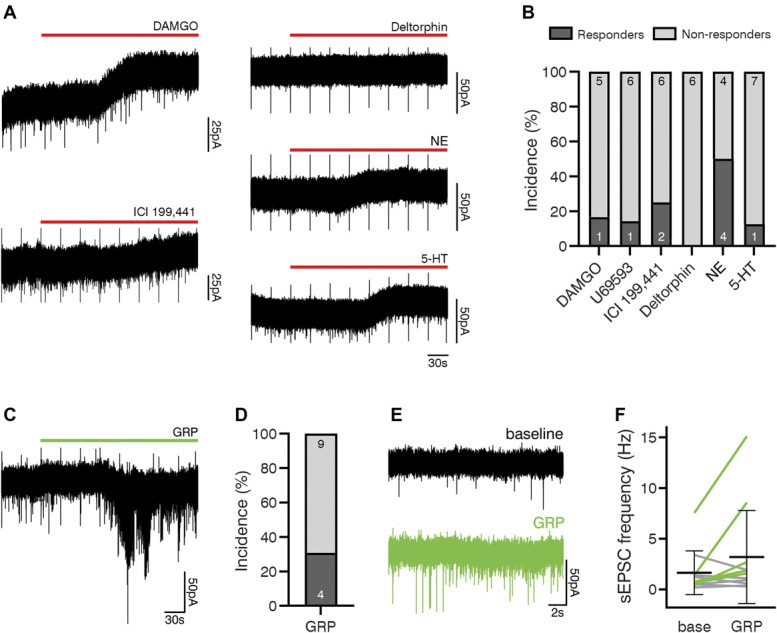
Responses of GRPR cells to neuromodulators and GRP. (A) Example traces demonstrate responses of GRPR cells to agonists for MOR (DAMGO), KOR (ICI 199,441), DOR (deltorphin), norepinephrinee (NE), and 5-HT. (B) These various agonists caused an outward current in GRPR cells as follows: DAMGO 1 of 6, U69593 1 of 7, ICI 199,441 2 of 8, NE 4 of 8, and 5-HT 1 of 8. Deltorphin did not cause a response in any of the 6 cells tested. (C) Responses of GRPR cells to the agonist, GRP, were also tested. Application of GRP resulted in an inward current (example in C) in 4 of 13 cells (D). GRP also resulted in an increase in sEPSC frequency in 5 of 11 cells, which included 2 cells that displayed an inward current in response to GRP and 3 that did not. In those cells that responded, GRP increased sEPSC frequency from 2.2 ± 3.0 Hz to 6.0 ± 5.9 Hz (F). The example sEPSC traces shown in (E) are taken from the recording illustrated in (C). In (F), green lines represent cells that responded to GRP and grey lines represent cells that did not. GRPR, gastrin-releasing peptide receptor; GFP, green fluorescent protein.

To investigate the effect of neuromodulators (opioids, NE, and 5-HT), various agonists were bath-applied to spinal cord slices (Figs. 10A and B). Only 1 GRPR cell (1/6, 16.7%) displayed an outward current in response to the MOR agonist DAMGO (3 µM), and the amplitude of which was 24.3 pA. None of the 6 cells tested responded to the DOR agonist [D-Ala^2^]-deltorphin II. Application of KOR agonists resulted in an outward current in 3 of 15 cells tested (2/8 for ICI 199,441, 6.1 and 13.95 pA, and 1/7 for U69593, 6.3 pA), consistent with coexpression of Grpr and Oprk1 in some cells^42^ and the proposed role of dynorphin acting on KORs to suppress itch^29,33^*.* Application of NE caused an outward current (14.8 ± 11.8 pA) in half of the cells tested (4/8, 50%). Only 1 cell (1/8, 12.5%) responded to 5-HT, which resulted in an outward current of 13.2 pA.

In addition to responses to opioids and monoamines, we also tested the responses of GRPR cells to GRP. Bath application of GRP caused an inward current (−15.5 ± 11.7 pA) in around a third of the cells tested (4/13, 30.8%) (Figs. 10C and D). GRP also increased sEPSC frequency, defined as a leftward shift in the distribution of interevent intervals, in almost half of cells tested (5/11, 45.5%). This included 2 cells that displayed a GRP-induced inward current and 3 that did not (Figs. 10E and F). In those cells that exhibited a change, GRP increased sEPSC frequency from 2.15 ± 3.04 to 5.96 ± 5.85 Hz. This phenomenon is likely to result from GRP causing increased action potential firing in other GRPR cells that were presynaptic to the recorded neuron, consistent with our anatomical finding that GRPR cells receive excitatory synaptic input from other GRPR cells.

### 3.9. Responses of gastrin-releasing peptide receptor cells to noxious and pruritic stimuli

**Figure 11.**
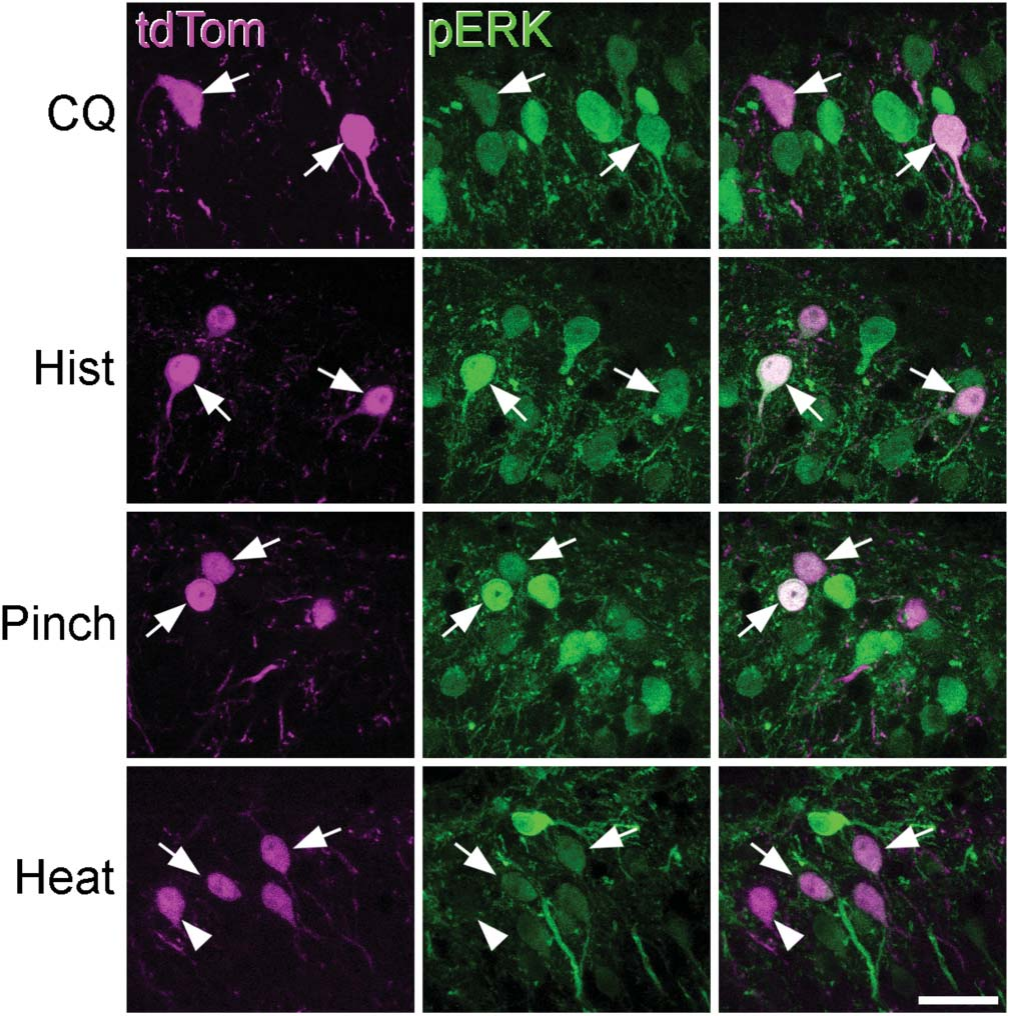
Phosphorylation of extracellular signal-regulated kinases (ERKs) in GRPR cells. Each row shows staining for tdTomato (tdTom, magenta) and phospho-ERK (pERK, green) in a section from a GRPR^CreERT2^;Ai9 mouse that had received intradermal injection of a pruritogen (either chloroquine [CQ] or histamine [Hist]) into the calf, or a noxious mechanical (pinch) or thermal (heat) stimulus. In each case, there are numerous pERK+ cells, while between 2 and 4 GRPR cells (labelled with tdTom) are visible. For chloroquine, histamine, and pinch, all of the tdTom+ cells are pERK+, and in each case, 2 are indicated with arrows. For the heat stimulus, 3 of the GRPR cells are pERK+ (2 shown with arrows), whereas 1 of the GRPR cells lacks pERK (arrowhead). Images are projections of 5 (CQ), 4 (Hist), 3 (pinch), and 4 (heat) optical sections with z-separations of 1 μm. Scale bar (for all parts) = 20 μm. GRPR, gastrin-releasing peptide receptor.

The distribution of pERK-positive cells seen after noxious or pruritic stimulation was similar to that reported in previous studies.^6,16,23^ In mice that had received intradermal injections of chloroquine or histamine 30 minutes before perfusion fixation, a cluster of pERK-positive cells was seen in laminae I and II of the L3 segment. These occupied a narrow mediolateral band in the middle part of the dorsal horn, which corresponds to the somatotopic representation of the injection site. As reported previously, few, if any, pERK-positive cells were seen in the animals injected with PBS that survived for 30 minutes.^6^ The pinch stimuli resulted in pERK-positive cells in the region of the SDH that receives input from the calf, whereas the distribution of pERK cells was broader for the noxious heat stimulus, consistent with the much larger region of the hind limb that was stimulated. Quantitative analysis was performed on tissue from the mice that had received noxious or pruritic stimuli by scanning the full thickness of 12 to 21 sections per animal. Examples of pERK and tdTomato staining are shown in Figure 11, and the result of the colocalisation analysis is provided in Table 4. For each of the noxious and pruritic stimuli, a very high proportion of the tdTomato cells within the activated region were pERK-positive. The proportions of tdTomato cells with pERK were 88% for pinch, 90% for heat, 89% for histamine, and 95% for chloroquine.

**Table 4:** Gastrin-releasing peptide receptor (GRPR) cells that were phosphorylated ERK (pERK)-positive after noxious or pruritic stimuli.

Stimulus	tdTom^+^ neurons	tdTom^+^ and pERK^+^	% tdTom neurons pERK^+^
Pinch	40 (34-46)	33 (30-35)	87.7% (69.6%-92.0%)
Noxious heat	90 (71-118)	81 (27-39)	89.6% (87.3%-92.7%)
Histamine	87 (64-111)	78 (63-103)	89.0% (85.6%-95.4%)
Chloroquine	80 (60-106)	76 (59-99)	95.3% (93.4%-96.7%)

Column 2 to 4 show the number of TdTomato+ (tdTom+) GRPR cells identified within the zone that contained pERK-positive cells, the number of TdTom+ and pERK double-labelled cells, and the proportion of TdTom+ cells with pERK, respectively. In each case, the mean is shown with the range in brackets.

### 3.10. Chemogenetic activation of gastrin-releasing peptide receptor cells

**Figure 12.**
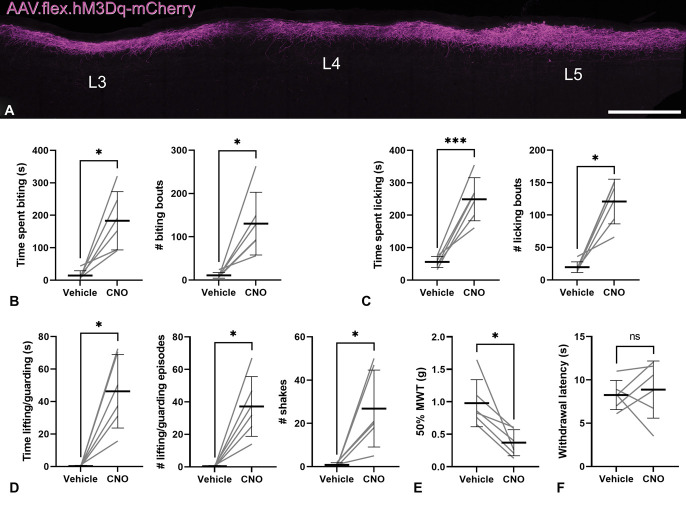
Chemogenetic activation of GRPR cells. (A) A sagittal section through the injection sites from one of the mice that received intraspinal injections of AAV.flex.hM3Dq-mCherry, targeted on the L3, L4, and L5 segments. The section has been stained to reveal mCherry (magenta). (B–F) Behavioural results seen after treatment with CNO or vehicle. In each case, plots show mean and standard deviation, whereas individual paired values for each mouse are shown with grey lines (n = 6). (B, C) Time spent biting or licking the affected area on the hind paw ipsilateral to the intraspinal injection site and number of biting or licking bouts. (D) Time spent lifting or guarding of the ipsilateral hind paw, number of lifting and/or guarding episodes, and the number of paw shakes. (E) Mean withdrawal threshold (MWT) as assessed with von Frey hairs. (F) Withdrawal latency to a radiant heat stimulus (Hargreaves test). Significance: *P < 0.05, **P < 0.01, ***P < 0.001, paired t tests. Scale bar in A = 500 μm. GRPR, gastrin-releasing peptide receptor.

Injection of AAV.flex.hM3Dq-mCherry into the L3 to L5 dorsal horn of GRPR^CreERT2^ mice resulted in a similar distribution of labelled cells to that seen after injection of either AAV.flex.eGFP or AAV.flex.tdTomato into this mouse line. mCherry-positive cells were largely restricted to the SDH and were present in the L3, L4, and L5 segments (Fig. 12A). The results of behavioural tests are given in Table 5 and shown Figures 12B–F and Figure S2 (available as supplemental digital content at http://links.lww.com/PAIN/B638). After injection with vehicle, mice displayed a low level of biting and licking of the hind limb, rump, and base of tail, both ipsilateral to the intraspinal injection (Figs. 12B–C) and on the contralateral side (data not shown). However, this did not differ significantly between the 2 sides and presumably reflects normal grooming behaviour. Consistent with a role for GRPR cells in itch, injection of 0.2 mg/kg CNO resulted in a greater than 10-fold increase in biting (both total duration and number of bouts) of the affected area in a 30-minute observation period (Table 5, Fig. 12B). However, we also noticed an increase in behaviours that are believed to be associated with pain. Licking of the affected area increased by >4-fold in total duration and by >6-fold in number of bouts after CNO (Table 5, Fig. 12C). After vehicle treatment, lifting and/or guarding or shaking of the paw was extremely rare events (<1 episode or occurrence in 30 minutes, with total duration of lifting and/or guarding < 1 second). However, these occurred relatively frequently after CNO administration, with a mean of 37 lifting and/or guarding episodes (46-second duration) and 27 paw shakes in 30 minutes (Table 5, Fig. 12D).

**Table 5:** Behavioural results from chemogenetic experiments.

		Vehicle	CNO	*P*	Statistical test
Behaviour					
Biting	Total duration (s)	14.4 ± 14.9	183.1 ± 89.6	0.0313	Wilcoxon
	Number of bouts	10.8 ± 6.6	130.3 ± 72.6	0.0313	Wilcoxon
Licking	Total duration (s)	56.2 ± 17.1	249.2 ± 66.4	0.0008	Paired t
	Number of bouts	19.7 ± 8.2	120.8 ± 34.4	0.0313	Wilcoxon
Lifting and/or guarding	Total duration (s)	0.3 ± 0.4	46.3 ± 22.6	0.0313	Wilcoxon
	Number of episodes	0.3 ± 0.5	37.2 ± 18.4	0.0313	Wilcoxon
Paw shaking	Number of occurrences	0.8 ± 1.0	26.8 ± 17.8	0.0177	Paired t

Values are expressed as mean ± SD.

A marked reduction in the mechanical withdrawal threshold was also observed after CNO injection, compared with vehicle (Fig. 12E; vehicle vs CNO = 0.98 ± 0.36 g vs 0.37 ± 0.20 g, mean ± S.D, *P* = 0.0138, paired *t* test), but no significant difference was seen in the withdrawal latency to radiant heat (Fig. 12F; vehicle vs CNO = 8.3 ± 1.7 seconds vs 8.9 ± 3.3 seconds, *P* = 0.6762, paired *t* test).

We have also looked for possible off-target effects by injecting CNO (5 mg/kg i.p.) into 8 wild-type mice that lacked DREADD receptors. This had no effect on von Frey or Hargreaves tests, and the animals showed no alteration in biting or licking behaviour (KAB, unpublished data). This strongly suggests that the effects we saw were mediated by CNO acting on hM3Dq expressed by the GRPR neurons.

## 4. Discussion

The main findings of this study are that (1) Cre-positive cells in the GRPR^CreERT2^ mouse account for ∼15% of excitatory neurons in the SDH and show little overlap with 5 of the 6 neurochemical populations that we previously identified in this region, (2) they can be classified morphologically as vertical cells, (3) their postsynaptic targets include other GRPR cells, (4) they respond to pruritic and noxious stimuli, and (5) activating them results in behaviours suggestive of both itch and pain.

### 4.1. Gastrin-releasing peptide receptor–expressing dorsal horn neurons

Previous studies of dorsal horn GRPR cells have used various genetically modified mouse lines. Aresh et al.^2^ generated a BAC transgenic GRPR::Cre line, crossed this with the Ai14 reporter, and found that 60% of tdTomato-positive cells in the SDH responded to GRP. However, although in situ hybridisation studies have shown that GRPR-expressing cells are concentrated in laminae I-IIo,^5^ most tdTomato cells in this cross were in laminae II-IV. This discrepancy may reflect ectopic expression of Cre or transient GRPR expression captured by the reporter. Several studies have used a BAC transgenic line (GRPR::GFP),^5,34,44^ in which GFP-labelled cells are concentrated in laminae I-IIo, matching the distribution of *Grpr* mRNA. However, although *Grpr* message is restricted to excitatory neurons,^54^ ∼20% of GFP cells in the GRPR::GFP line are inhibitory.^34,44^ The proportions of GFP cells found to respond to application of GRP varied from ∼70%^5^ to 100%,^34^ although Pagani et al.^44^ reported that while all excitatory GFP cells responded, none of the inhibitory ones did. Two recent studies used a mouse in which iCre was knocked into the GRPR locus,^13,42^ and the distribution of labelled cells seen after intraspinal injection of AAV coding for Cre-dependent yellow fluorescent protein was similar to that seen in this study.

Our in situ hybridisation findings confirm excellent correspondence between *iCre* and *Grpr* mRNAs in the GRPR^CreERT2^ mouse line,^41^ and it is therefore surprising that only ∼one-third of these cells responded to GRP. It is possible that some cells lack appropriate second messenger pathways or that these were disrupted by patching the cell. However, an alternative explanation is that in many cells the level of GRPR protein in the membrane was too low to result in a detectable response to GRP. This may be less of an issue with the GRPR::GFP line because cells with strong GFP labelling are likely to be selected for patching, and these may have relatively high levels of the receptor. In the GRPR^CreERT2^ line, Cre acts as a switch by deleting a STOP cassette or inverting an antisense sequence, and the expression level for the resulting transgene is unlikely to match that of GRPR itself.

Although some neurons captured in the GRPR^CreERT2^ mouse may not express functional levels of the receptor, our findings that these cells are largely restricted to the SDH, that they are morphologically homogeneous, and that they show little overlap with the neurotensin, CCK, NKB, NPFF, or GRP–GFP neurons suggest that they represent a distinct population. The overlap with Tac1 cells is likely to reflect the broader expression of *Tac1* mRNA because this is found not only in a population of radial cells in lamina II^16^ but also in some inhibitory interneurons^24,27^ and in many lamina I projection cells.^30,47^ Consistent with this suggestion, we have found that some Tac1-expressing cells, identified with the viral Brainbow approach, show vertical morphology (MG-M, EP, and AJT, unpublished observations).

### 4.2. Gastrin-releasing peptide receptor expression by vertical cells

Grudt and Perl^22^ used the term “vertical cells” to describe a population of lamina II neurons. However, we found that GRPR cells in both laminae I and IIo invariably had prominent ventral dendrites, resembling vertical cells described in other studies. We have therefore extended this term to cover lamina I neurons with similar morphology. Our finding that the GRPR neurons correspond to vertical cells is consistent with that of Koga et al.,^34^ who reported that 65% of patched cells in the GRPR::GFP line belonged to this class. The lower proportion seen in their study probably results (at least partly) from the capture of inhibitory neurons in this line. Many of the GRPR cells showed delayed firing, consistent with previous reports for both GRPR neurons^5,34,44^ and vertical cells.^9,22,51,67^ The single-spike pattern, which we found in ∼40% of cells, has seldom been seen in either of these classes. However, firing patterns depend on the recording conditions, and simulations have shown that delayed and single-spike firing can arise from similar ion channel densities.^3^

Previous studies have reported that most vertical cells receive monosynaptic primary afferent input, arising from Aδ and/or C afferents.^22,66^ By contrast, we could only confirm monosynaptic input in 25% of cases, and this was invariably from C fibres. However, we are likely to have underestimated the proportion of GRPR cells receiving monosynaptic input for technical reasons, such as transection of afferents during slice preparation, or failure to stimulate the appropriate dorsal roots. Consistent with this interpretation, we found that 7 of 10 cells showed increased mEPSC frequency in the presence of capsaicin, indicating that they received direct input from TRPV1+ afferents. Interestingly, this differs from the findings of Zheng et al.,^70^ who saw no change in mEPSC frequency when capsaicin was applied during recordings from vertical cells. Another difference from previous findings is that only 1 of 8 GRPR cells responded to 5-HT, and only half responded to norepinephrine, whereas Lu and Perl^38^ found that all of their vertical cells showed outward currents in response to both monoamines. One possible reason for these discrepancies is that they reflect species differences because some of these previous studies were performed on guinea pig^22^ or rat.^38,66^ However, an alternative explanation is that there are other types of vertical cells, and that these were included in previous studies. In support of this, we have recently found that neurons belonging to the NPFF population are also vertical cells (RQ, EP, MGM, and AJT, unpublished data). In addition, we previously reported that some dynorphin-expressing excitatory interneurons were vertical cells,^29^ and these do not overlap with the GRPR population (MGM and AJT, unpublished observations).

Lu and Perl^37^ reported that transient central cells were often presynaptic to vertical cells, and it was subsequently suggested that this connection forms part of a circuit for tactile allodynia.^36^ Interestingly, recent reports have identified GRP–GFP neurons as transient central cells,^16,44^ and Pagani et al. showed that these provide synaptic input to the GRPR neurons as part of a putative itch pathway.^44^ It is therefore possible that the transient central to vertical cell circuit identified by Lu and Perl corresponds to this GRP–GFP to GRPR cell pathway and thus contributes to perception of itch.

### 4.3. Role for gastrin-releasing peptide receptor cells in both itch and pain

Although GRPR cells are believed to act as third-order neurons in the itch pathway (“tertiary pruritoceptors”),^29,40,44^ our results indicate that many of them are directly innervated by Trpv1-expressing (nociceptive) primary afferents, and consistent with this, the majority responded to noxious stimulation. We also found that chemogenetically activating the GRPR cells evoked pain-related and itch-related behaviours. Interestingly, activation of GRP-expressing neurons (which form glutamatergic synapses on the GRPR cells^44^) was also found to result in both pain and itch behaviour.^56^ It is therefore likely that the GRPR cells captured in the GRPR^CreERT2^ line convey both nociceptive and pruritoceptive information to ALS projection neurons.

At first sight, this is at odds with the finding that intrathecal administration of GRP causes itch, but not pain, behaviour,^33,45,57^ and that GRPR knockout, or ablation of GRPR-expressing cells, selectively suppresses itch.^58^ Because we found that only some of the neurons captured in this line responded to GRP, one possible explanation is that these represent a functionally distinct subset that exclusively convey itch, whereas the remaining (nonresponsive) cells contribute to pain processing. However, this seems unlikely because ∼90% of the cells labelled in the GRPR^CreERT2^ mouse responded to noxious stimuli. A more likely explanation is that GRP-GRPR signalling is itch-specific, whereas other signalling pathways used by the GRPR cells (eg, glutamatergic input from nociceptive afferents and GRP-expressing excitatory interneurons) are involved in pain. Ablation of GRPR-expressing cells may preferentially affect those with relatively high levels of expression and therefore selectively prevent itch. Alternatively, this selectivity may reflect a form of redundancy, in which transmission of nociceptive information by other pathways is sufficient to evoke normal pain behaviours, even when the GRPR cells have been ablated.

Vertical cells are known to innervate lamina I projection neurons,^15,37^ and consistent with this, Mu et al.^41^ demonstrated synaptic input from GRPR cells to some spinoparabrachial neurons in this lamina. However, we find that axons of the GRPR cells also target the lateral part of lamina V and the adjacent white matter, areas that contains many wide-dynamic-range ALS neurons.^65^ The GRPR cells may therefore also provide a route through which nociceptive information can reach these deep projection cells.

## Conflict of interest statement

The authors have no conflicts of interest to declare.

## Appendix A. Supplemental digital content

Supplemental digital content associated with this article can be found online at http://links.lww.com/PAIN/B638.

## Supplemental video content

A video abstract associated with this article can be found at http://links.lww.com/PAIN/B639.
