# Supplementary material for: Grpr expression defines a population of superficial dorsal horn vertical cells that have a role in both itch and pain
Source: Pain. 2022 May 11;164(1):149–70. doi: 10.1097/j.pain.0000000000002677 (PMC9756441; doi:10.1097/j.pain.0000000000002677)
Supplement: Supplementary file 2 [file jop-164-149-s002.pdf]

Fig S1 Comparison of electrophysiological properties of the GRPR cells with those that express substance P (SP) and GRP-GFP cells. Data for the SP and GRP-GFP was obtained from Dickie et al [16]. Bars represent mean ± SD. *P<0.05, **P<0.01, ****P<0.0001, Kruskal-Wallis test, followed by Dunn's multiple comparison test.

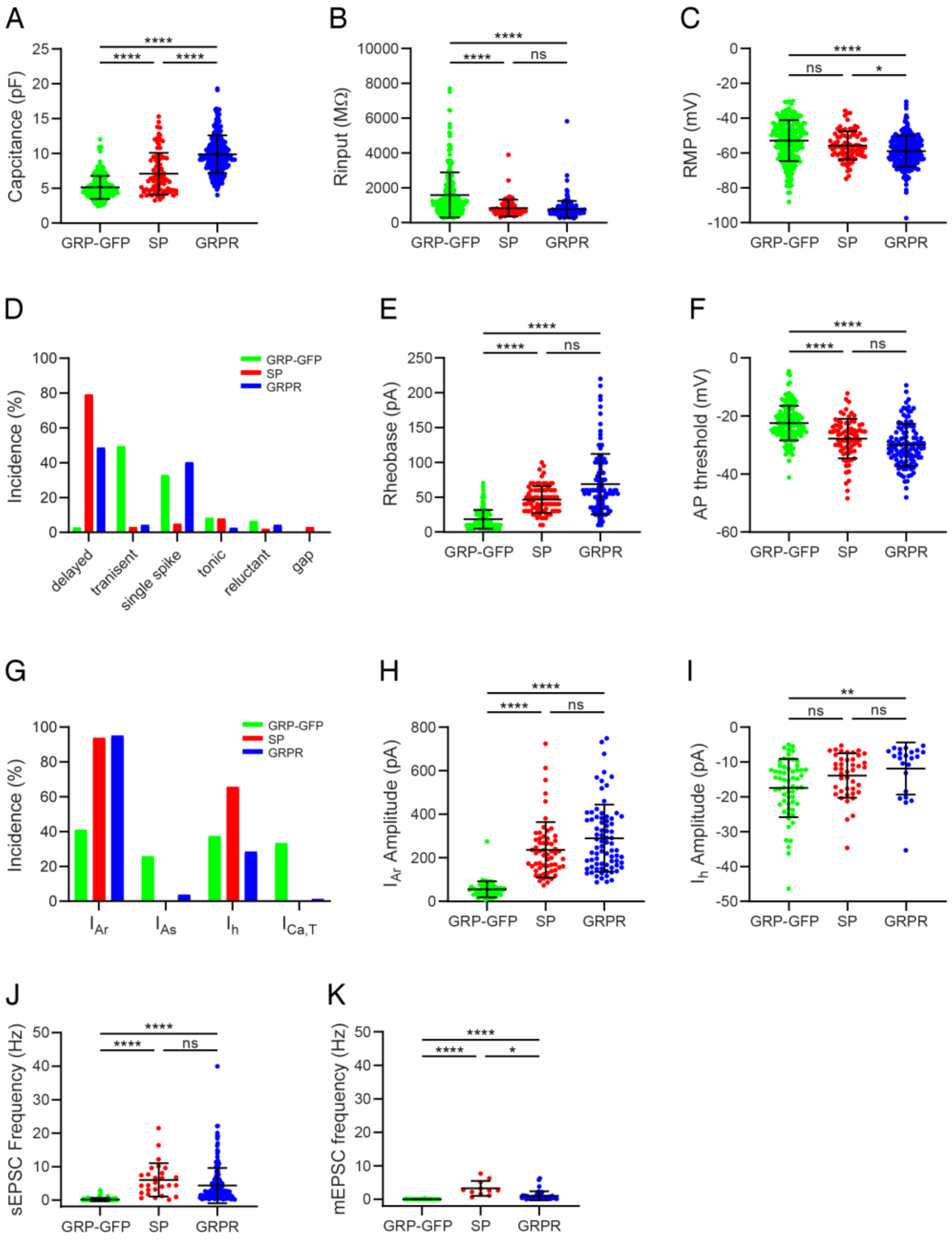

Fig S2 Behavioural response to chemogenetic activation of GRPR cells. Raster plots from a representative animal showing bouts of biting and licking within ipsilateral L3-5 dermatomes, as well as episodes of lifting/guarding and occurrences of shaking of the ipsilateral paw over 30 minutes immediately following injection with vehicle or 0.2mg/kg CNO.

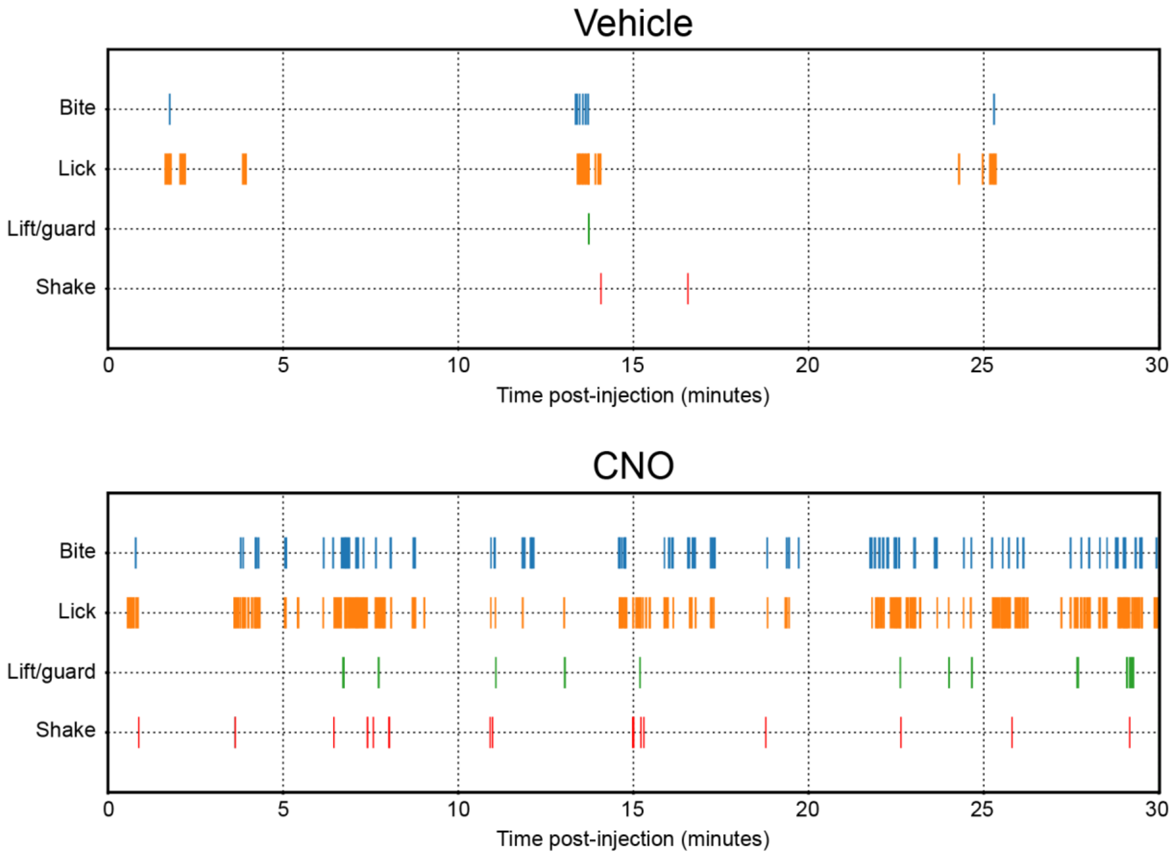

Table S1 Comparison between electrophysiological properties of GRPR, Substance P and GRP-GFP cells.

	GRPR	SP	GRP-GFP	Significance	
				GRPR vs SP	GRPR vs GRP-GFP
Capacitance	9.88 ± 2.7 pF (n = 256)	7.07 ± 3.0 pF (n = 85)	5.12 ± 1.6 pF (n = 232)	P <0.0001	P <0.0001
Input resistance	767 ± 473 MΩ (n = 223)	836 ± 481 MΩ (n = 84)	1588 ± 1297 MΩ (n = 232)	P = 0.713	P <0.0001
Resting membrane potential	-58.9 ± 8.9 mV (n = 218)	-55.8 ± 8.1 mV (n = 84)	-52.9 ± 11.8 mV (n = 230)	P = 0.046	P <0.0001
Rheobase	68.7 ± 43.4 pA (n = 111)	46.5 ± 19.2 pA (n = 80)	18.3 ± 13.4 pA (n = 155)	P = 0.065	P <0.0001
Action potential threshold	-30.0 ± 7.3 mV (n = 111)	-27.8 ± 6.8 mV (n = 80)	-22.5 ± 6.0 mV (n = 155)	P = 0.054	P <0.0001
Action potential latency	349.5 ± 380.0 ms (n = 111)	595.0 ± 252.8 ms (n = 80)	137.1 ± 77.6 ms (n = 155)	P <0.0001	P = 0.341
Action potential width	1.4 ± 0.4 ms (n = 111)	3.5 ± 1.4 ms (n = 80)	2.4 ± 1.6 ms (n = 155)	P <0.0001	P <0.0001

Action potential height	45.2 ± 10.4 mV (n = 111)	47.5 ± 10.5 mV (n = 80)	49.4 ± 12.9 mV (n = 155)	P = 0.764	P = 0.009
After-hyperpolarisation	-25.9 ± 4.9 mV (n = 111)	25.2 ± 5.0 mV (n = 80)	-32.9 ± 7.6 mV (n = 155)	P >0.999	P <0.0001
I_{Ar} amplitude	289.7 ± 154.7 pA (n = 77)	263.3 ± 127.4 pA (n = 60)	54.9 ± 36.7 pA (n = 65)	P = 0.427	P <0.0001
I_h amplitude	-11.9 ± 7.5 pA (n = 23)	-13.9 ± 6.4 pA (n = 42)	-17.5 ± 8.4 pA (n = 59)	P = 0.385	P = 0.002
sEPSC frequency	4.34 ± 5.26 Hz (n = 189)	6.04 ± 4.98 Hz (n = 27)	0.20 ± 0.50 Hz (n = 120)	P = 0.317	P <0.0001
mEPSC frequency	1.02 ± 1.34 Hz (n = 42)	3.24 ± 2.21 Hz (n = 11)	0.02 ± 0.06 Hz (n = 32)	P = 0.041	P <0.0001

Data for the SP and GRP-GFP cells are taken from [16]
